# A systematic review of automated methods to perform white matter tract segmentation

**DOI:** 10.3389/fnins.2024.1376570

**Published:** 2024-03-19

**Authors:** Ankita Joshi, Hailong Li, Nehal A. Parikh, Lili He

**Affiliations:** ^1^Imaging Research Center, Department of Radiology, Cincinnati Children's Hospital Medical Center, Cincinnati, OH, United States; ^2^Neurodevelopmental Disorders Prevention Center, Perinatal Institute, Cincinnati Children's Hospital Medical Center, Cincinnati, OH, United States; ^3^Department of Radiology, University of Cincinnati College of Medicine, Cincinnati, OH, United States; ^4^Department of Pediatrics, University of Cincinnati College of Medicine, Cincinnati, OH, United States; ^5^Computer Science, Biomedical Informatics, and Biomedical Engineering, University of Cincinnati, Cincinnati, OH, United States

**Keywords:** diffusion magnetic resonance imaging (dMRI), white matter tract, segmentation, systematic review, tract segmentation, tractography

## Abstract

White matter tract segmentation is a pivotal research area that leverages diffusion-weighted magnetic resonance imaging (dMRI) for the identification and mapping of individual white matter tracts and their trajectories. This study aims to provide a comprehensive systematic literature review on automated methods for white matter tract segmentation in brain dMRI scans. Articles on PubMed, ScienceDirect [NeuroImage, NeuroImage (Clinical), Medical Image Analysis], Scopus and IEEEXplore databases and Conference proceedings of Medical Imaging Computing and Computer Assisted Intervention Society (MICCAI) and International Symposium on Biomedical Imaging (ISBI), were searched in the range from January 2013 until September 2023. This systematic search and review identified 619 articles. Adhering to the specified search criteria using the query, “*white matter tract segmentation* OR *fiber tract identification* OR fiber *bundle segmentation* OR *tractography dissection* OR *white matter parcellation* OR *tract segmentation,”* 59 published studies were selected. Among these, 27% employed direct voxel-based methods, 25% applied streamline-based clustering methods, 20% used streamline-based classification methods, 14% implemented atlas-based methods, and 14% utilized hybrid approaches. The paper delves into the research gaps and challenges associated with each of these categories. Additionally, this review paper illuminates the most frequently utilized public datasets for tract segmentation along with their specific characteristics. Furthermore, it presents evaluation strategies and their key attributes. The review concludes with a detailed discussion of the challenges and future directions in this field.

## 1 Introduction

The development of diffusion magnetic resonance imaging (dMRI) coupled with the subsequent introduction of techniques to model water diffusion within the brain tissue using a diffusion tensor model (DTI) (Delmarcelle and Hesselink, 1992; Basser et al., 1994a, 1994b; Carter et al., 2015), has led to unprecedented opportunities for noninvasive exploration of the brain’s intricate white matter (WM) structures (Clayden et al., 2007). Tractography is a technique that harnesses data derived from dMRI to reconstruct and visualize the WM pathways within the brain by tracing the likely paths of water diffusion. Tractography (Mori et al., 1999; Basser et al., 2000) involves the algorithmic reconstruction of these WM pathways, generating a multitude of fibers (El Kouby et al., 2005) for each subject. This is followed by the delineation of the obtained fiber trajectories or streamlines into bundles or their association with anatomically well-defined tracts, a process commonly referred to as WM tract segmentation or dissection (Bullock et al., 2019).

WM tracts in the brain serve as the communication highways that connect different regions of the brain. Accurate segmentation enables researchers and clinicians to identify specific tracts associated with particular neurological functions, including cognitive, motor, and behavioral processes (Yushkevich et al., 2008; Sadeghi et al., 2013). Accurate tract segmentation plays a pivotal role in comprehending alterations in the micro- and macro-structure of the brain’s WM. It enhances our understanding of how structural connectivity shapes brain function and development. Additionally, it provides valuable insights into neurological diseases, including cognitive impairment and neurodegeneration, mental health disorders, and the aging process (Catani, 2006; De Belder et al., 2012; Le Bihan and Johansen-Berg, 2012). Moreover, accurate WM tract segmentation holds immense clinical significance, particularly in aiding in pre-operative and intra-operative brain tumor resections. It facilitates the visualization and localization of WM tracts that may be displaced or affected by tumors (Lazar et al., 2006; Chen et al., 2016; Essayed et al., 2017; Vanderweyen et al., 2020). It is worth noting that WM tract segmentation is a very challenging task. The human brain contains millions of intertwined axonal pathways, and these fibers can cross, split, or merge, making it challenging to accurately track individual pathways.

Most techniques employed for WM tract segmentation are based on virtual dissection or manual approaches, which involve the meticulous delineation of regions of interest (ROIs) (Catani et al., 2002; Mori and van Zijl, 2007; Wakana et al., 2007). These ROIs define where streamlines should pass and where streamlines should terminate. The provision of ROIs requires expert knowledge and hence manual methods incur expert labor costs. Manual methods face practical challenges in their adoption since they are time-consuming and expensive due to their high clinical and labor costs. Nevertheless, manual methods remain the gold standard for delineating WM tracts and serve as a critical benchmark for validating alternative approaches. The advent of better imaging techniques, improved image quality and higher resolutions (Van Essen et al., 2012), along with the application of sophisticated post-processing techniques, has driven a significant surge in the development of automated methods for tract segmentation (Yamada et al., 2009; Essayed et al., 2017; Ghazi et al., 2023).

A wide range of automated white matter tract segmentation methods have been developed over the years. While multiple works exist that review tractography methods and their applications, currently, there is limited literature available that specifically discusses the topic of delineating white matter tracts. Authors summarize the various categories that tractography segmentation methods fall under (Zhang et al., 2022) when reviewing quantitative tractography methods for studying the brain’s structural connectivity in health and disease. Recently, authors in Ghazi et al. (2023) have reviewed literature focusing on deep learning approaches for tract segmentation. In this work, we extend the scope by conducting a systematic and comprehensive review of automated approaches for the segmentation of white matter tracts in the last decade. This paper contributes to the following:

1. Review of automated tract segmentation methods explored within the last 10 years with respect to key research questions.
2. Identify the categories of methods and their research gaps and challenges.
3. Highlight an overview of the various datasets and evaluation metrics used in the methods.
4. Discuss the future directions that can be conducted.

The remainder of the survey is organized as follows: Section 2 presents the review planning, Section 3 introduces the key findings as results, Section 4 summarizes and discusses the findings, Section 5 outlines the future directions, and Section 6 concludes the review.

## 2 Review planning

This section is dedicated to planning the review: the comprehensive research questions related to the study are rigorously defined, the identification criteria and the resources of study are detailed.

### 2.1 Key research questions

- What method is developed?
- What dataset is used?
- What evaluation metrics are used?
- What category of method does the study fall under?
- Is the code for the automatic tract segmentation method publicly available, is the practical applicability of the method discussed in terms of computation time and external validation?

### 2.2 Sources of information

The sources of information listed below were searched between the time span from *January 2013 until September 2023* using the query “*white matter tract segmentation* OR *fiber tract identification* OR fiber *bundle segmentation* OR *tractography dissection* OR *white matter parcellation* OR *tract segmentation”*

- Pubmed (https://pubmed.ncbi.nlm.nih.gov/)
- Science direct (https://www.sciencedirect.com) for publication titles under NeuroImage, NeuroImage: Clinical, and Medical Image Analysis
- Scopus (https://www.scopus.com/)
- IEEE explore digital library (https://ieeexplore.ieee.org/)
- Conference publications for: Medical Imaging Computing and Computer Assisted Intervention Society (MICCAI), International Symposium on Biomedical Imaging (ISBI)

### 2.3 Inclusion criteria

Inclusion requirements were: (a) original research article published in the selected journal publications of Pubmed, ScienceDirect, Scopus, IEEE Explore Digital library and conference publications MICCAI and ISBI; (b) published within the last 10 years from January 2013 until September 2023; (c) published in English; (d) performed automated white matter tract segmentation in human brains; and (e) research articles specifically developing automated methods for white matter tract segmentation performed on deep white matter. Search strings were established via literature search and domain expertise. Specifically, title and abstract articles were searched on each of the above-mentioned sources of information using strings: white matter tract segmentation OR fiber tract identification OR fiber bundle segmentation OR tractography dissection OR white matter parcellation OR tract segmentation.

## 3 Results

**Figure 1:**
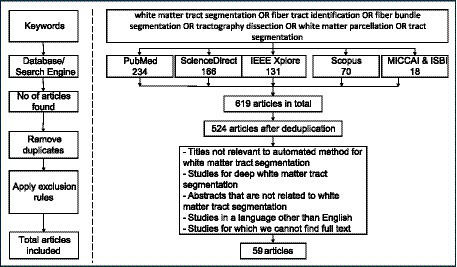
Flow diagram for articles retrieved in this study.

Our search strategy retrieved 619 articles published between January 2013 and September 2023. After articles were reviewed for definite exclusions and the bibliography of eligible articles were hand-searched, 59 articles met the inclusion criteria. Figure 1 shows the flow diagram of the retrieved articles and the rules applied to get the resulting 59 articles. The results are presented as follows: First, we summarize the major datasets used in the studies included in this review. We then provide a list of the 59 research articles by focusing on the research questions established. These research articles are mentioned according to the categories they belong to and finally we provide a summary for the evaluation metrics used by the studies.

### 3.1 Datasets

We present a list of the most commonly used imaging datasets used for the 59 studies. For each dataset we highlight the population details, the MRI acquisition details and online link to access the dataset. Table 1 lists the dataset studied.

**Table 1:** Summary of the datasets used in the papers included in this review.

**Dataset**	**Online Link**	**Subjects**	**MRI details**
Human Connectome Project (HCP) (Van Essen et al., 2013)	https://humanconnectome.org	1,200 healthy young adults between ages 22–35 years	1.25 mm3 isotropic resolution, 270 gradient directions with 3 b-values (1,000,2000,3,000 s/mm^2^) and 18 b − 0 images
Developing Human Connectome Project (dHCP) (Makropoulos et al., 2018)	www.developingconnectome.org	783 healthy newborn babies between postmenstrual ages ranging from 26 to 45 weeks	1.5 mm × 1.5 mm × 3 mm resolution; uniform distributed set of directions on 4 shells *b* = 0 s/mm^2^:20, *b* = 400 s/mm^2^: 64, *b* = 1000s/mm^2^:88, *b* = 2,600 s/mm^2^:128; TR/TE = 3800/90 ms
Consortium for Neuropsychiatric Phenomics (CNP) (Poldrack et al., 2016)	http://openfmri.org	130 subjects, healthy and patient (ADHD, bipolar disorder, schizophrenia) groups between 21 and 50 years	2 mm^3^ isotropic resolution; 64 directions; TR/TE = 9000/93 ms; *b* = 1,000 s/mm^2^
Multiple Acquisitions for Standardization of Structural Imaging Validation and Evaluation (MASSIVE) (Froeling et al., 2017)	www.massive-data.org	8,000 unique dMRI volumes acquired of a single healthy subject	2.5mm^3^ isotropic resolution, multiple shells of 125, 250, 250, 250, and 300 gradient orientations, and *b*-values of 500, 1,000, 2000, 3,000, and 4,000 s/mm^2^ respectively, additional 204 *b* = 0 s/mm^2^ images
Autism Brain Imaging Data Exchange (ABIDE) (Di Martino et al., 2017)	http://fcon_1,000.projects.nitrc.org/indi/abide/	subjects between 5 and 17 years of age	resolution of 3mm^3^ *b* = 1000s/mm^2^; 64 directions; TR/TE = 5200/78 ms
Rotterdam Study (Hofman et al., 2015)	https://www.ergo-onderzoek.nl/	9,752 dMRI scans from 5,286 participants with mean age 64.7 ± 9.9 years	imaging matrix of 64 × 96 zero-padded in k-space to 256 × 256 in a field of view of 210 × 210 mm^2^, TR/TE = 8575/82.6 ms, 25 diffusion weighted volumes along non-colinear directions using a b-value of 1000s/mm^2^
Non-invasive Exploration of brain connectivity and Tracts (CONNECT/ARCHI) (Schmitt et al., 2012)	https://www.humanbrainproject.eu/ and ARCHI database can be requested from cyril.poupon@cea.fr	79 healthy subjects, age between 18 and 40 years	1.71875 × 1.71875 × 1.7 mm resolution, 60 optimized diffusion directions *b* = 1,500 s/mm^2^, one *b* = 0 image, TR/TE = 14,000/93 ms
Growing Up in Singapore Toward Health Outcomes (GUSTO) study (Soh et al., 2014)	http://www.gusto.sg/	388 neonates screened at day 7, 30 at 6 weeks, and/or 50 babies screened at 6 months since birth.	TR /TE = 7000/56 ms; flip angle = 90°; FOV = 200 mm × 200 mm; matrix size = 256 × 256; 19 images with *b* = 600 s/mm^2^ and 1 with *b* = 0 s/mm^2^
Parkinson’s Progression Markers Initiative (PPMI) (Marek et al., 2011)	https://www.ppmi-info.org	400 recently diagnosed of Parkinson disease and 200 healthy subjects	2 mm^3^ isotropic resolution; 64 directions; TE/TR = 7600/88 ms; *b* = 1,000 s/mm^2^
Adolescent Brain Cognitive Development (ABCD) (Volkow et al., 2018)	https://abcdstudy.org/	10,000 children starting at 9–10 years up to ages 19–21	1.7 mm^3^ resolution; 96 directions; TR/TE = 4100/88 ms; *b* = 3,000 s/mm^2^

For each study, we give the online availability of the dataset, the population details involved in the study and the MRI acquisition details.

### 3.2 Automated methods for white matter tract segmentation

**Figure 2:**
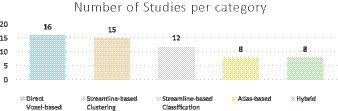
The above chart shows the number of studies included in each category.

All automated methods included in this review can be classified into categories based on the specific technique used for automatic tract segmentation. The high-level categories have been specified in Table 2, noting the references in which they were implemented. Figure 2 shows a bar graph of the distribution of studies within the categories. Some studies have used methods which have been developed as a combination of multiple categories and are referred to as a hybrid approach. The goal of this section is to investigate the findings corresponding to the questions framed in the review planning phase in Section 2. Tables 3–7 give a list of each of the studies and summarizes their inclusion criteria; the dataset used in the study, an overview of the approach used, the evaluation metrics used to validate the results in the work, and finally the practical application of the study in terms of public availability of the algorithm, the computational runtime to segment white matter tracts for a single subject and whether external validation has been conducted.

**Table 2:** Categories of methods identified in this review and the corresponding studies included.

**Category**	**References**
Direct voxel-based	Ocegueda and Rivera (2013), Wasserthal et al. (2018, 2019), Dong et al. (2019), Pomiecko et al. (2019), Lu et al. (2020, 2021, 2022), Nelkenbaum et al. (2020), Li et al. (2021), Liu et al. (2022, 2023), Lucena et al. (2022), Wang et al. (2022), and Yin et al. (2022)
Streamline-based clustering	Tunc et al. (2014), Jin and Cetingül (2015), Kamali and Stashuk (2016), Kumar and Desrosiers (2016), Gupta et al. (2017, 2018), Roman et al. (2017), Siless et al. (2018), Vázquez et al. (2020), Yang et al. (2020), Chen et al. (2021, 2023), Xu et al. (2021), Logiraj et al. (2021a), and Zhao et al. (2022)
Streamline-based classification	Ratnarajah and Qiu (2014), Heker et al. (2016), Ngattai Lam et al. (2018), Bertò et al. (2019, 2021), Liu et al. (2019), Ugurlu et al. (2019), Zhang et al. (2019, 2020), Wu et al. (2020), Logiraj et al. (2021b), and Dumais et al. (2023)
Atlas-based	Jin et al. (2013), Yoo et al. (2015), Labra et al. (2017), Sharmin et al. (2018), Zhang et al. (2018), Vázquez et al. (2019), Jordan et al. (2021), and Radwan et al. (2022)
Hybrid	Wassermann et al. (2013, 2016), Chekir et al. (2014), O’Donnell et al. (2017), Garyfallidis et al. (2018), Delmonte et al. (2019), Peretzke et al. (2023), and Xu et al. (2023)

**Table 3:** Direct voxel-based methods for Automated White Matter Tract Segmentation.

**Direct Voxel-based methods**									
**Author/Year/Citation**	**Dataset/No. of Subjects**	**Main Context**	**Architecture**	**No. of tracts segmented**	**Data Augmentation**	**Performance Metrics**	**Practical Application**		
							**Code**	**Runtime per subject**	**External validation**
Liu, Wan, et al./2023/ (Liu et al., 2023)	HCP/105; Private/16	- Transfer knowledge of pretrained CNN using fine-tuning strategy for new tracts with only a single annotated scan - Use extensive data augmentation	2D U-net (Ronneberger et al., 2015)	12	Random Cutout, Tract Cutout	Dice: 0.619 ~ 0.693	N/A	N/A	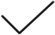
Lucena, Oeslle, et al./2022/ (Lucena et al., 2022)	HCP/105	- Based on 3D nnUNet with raw dMRI intensities transformed into spherical harmonics (SH) space - Also output uncertainty measurement with respect to groundtruth	3D nnUNet (Isensee et al., 2021)	72	3D rotation to both the spatial location and SH coefficients	Dice: 0.82 Sensitivity: 0.85 ~ 0.86 Specificity: 0.78 ~ 0.80 ASSD: 0.63 ~ 0.66 Hausdorff distance: 9.24 ~ 10.57	https://github.com/OeslleLucena/TractSegmentation (Link inactive)	N/A	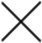
Lu, Qi, et al./2022/ (Lu et al., 2022)	HCP/100; Private/12	- Transfer knowledge of pretrained CNN using fine-tuning strategy for new tracts with only few annotated scans - Utilize data augmentation strategy for learning in few-shot setting	2D U-net (Ronneberger et al., 2015)	12	Mixing-based data augmentation (Zhang et al., 2017; Yun et al., 2019)	Dice: 0.780 ~ 0.846 Relative Volume Difference (RVD): 0.129 ~ 0.156	N/A	N/A	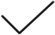
Liu, Wan, et al./2022/ (Liu et al., 2022)	HCP/100; Private/17	- Utilize tract correlation by embedding tract labels as a vector - Integrate label embedding with segmentation module built using TractSeg (Wasserthal et al., 2018)	2D U-net (Ronneberger et al., 2015)	72	Angular and spatial downsampling of dMRI	Dice: 0.582 ~ 0.851	https://github.com/liuwan0208/TractSegWithLabelEmbedding	N/A	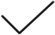
Wang, Zhenwei, et al./2022/ (Wang et al., 2022)	HCP/205 3D Fiber atlas (Zhang et al., 2018)	- Represent the spatial distribution and shape of fibers using a novel descriptor called FiberGeoMap	Transformer (Vaswani et al., 2017)	103	N/A	Precision: 0.9279 Recall: 0.9478 Accuracy: 0.9319 Dice: 0.9268	https://github.com/Garand0o0/FiberTractSegmentation	N/A	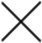
Yin, Haoran, et al./2022/ (Yin et al., 2022)	HCP/105	- Utilized a modified U-net architecture to use a dense crisscross attention mechanism	CCNet (Huang et al., 2023)	72	Elastic Deformation, rotation, resampling, gaussian noise, displacement, zooming	Dice: 0.843	N/A	N/A	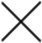
Lu, Qi, Yuxing Li, and Chuyang Ye/ 2021/ (Lu et al., 2021)	HCP/155	- Exploit self-supervised learning since pretext tasks do not require manual annotations - Transfer knowledge learned in pretraining using fine-tuning	2D U-net (Ronneberger et al., 2015)	72	Angular and spatial downsampling of dMRI	Dice: 0.813 RVD: 0.128	N/A	N/A	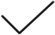
Li, Siqi, et al./2021/ (Li et al., 2021)	HCP/102	- Utilize fractional anisotropy (FA) images and T1 weighted images - combine output of two parallel architectures for final output	2D U-net (Ronneberger et al., 2015)	1	Cropping, Contrast augmentation, Brightness augmentation, Hue augmentation	Dice: 0.855	N/A	N/A	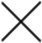
Lu, Qi, Yuxing Li, and Chuyang Ye/2020/ (Lu et al., 2020)	HCP/155	- Exploit self-supervised learning along with pseudo-labelling	2D U-net (Ronneberger et al., 2015)	72	N/A	Dice: 0.761 ~ 0.768	N/A	N/A	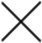
Li, Bo, et al./2020/ (Li et al., 2020)	Rotterdam Study/5286 Iris Study (Steketee et al., 2016)/−	- Utilize 4D diffusion tensor image directly as input - Separate network trained for each tract	3D U-net	25	N/A	Dice: 0.72 ~ 0.83	N/A	0.49 s	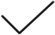
Nelkenbaum, Ilya, et al./2020/ (Nelkenbaum et al., 2020)	HCP/105	- Utilize both T1-weighted and principal direction of diffusion (PDD) images as input	VNet (Milletari et al., 2016)	14	Angular and spatial downsampling of dMRI	Dice: 0.722 ~ 0.869	N/A	N/A	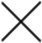
Wasserthal, Jakob, et al./2019/ (Wasserthal et al., 2019)	HCP/105	- Built on top of TractSeg (Wasserthal et al., 2018) - Module for tract start and end segmentation added – Module for tract orientation mapping (TOM) prediction added	2D U-net (Ronneberger et al., 2015)	72	Rotation, Elastic deformation, Displacement, Zooming, Resampling, Gaussian noise	Dice: 0.74 ~ 0.85	https://github.com/MIC-DKFZ/TractSeg/	8.95 min	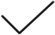
Pomiecko, Kristofer, et al./ 2019/ (Pomiecko et al., 2019)	Private/240	- Utilize whole brain MRI diffusion anisotropy maps as input - Separate network trained for each tract	Multi-scale 3D U-net based on DeepMedic (Kamnitsas et al., 2017)	12	N/A	Dice: 0.72	N/A	16 s	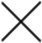
Dong, Xiaofeng, et al./2019/ (Dong et al., 2019)	HCP/105 Human Brain Data Sharing Initiative (HBDSI)/−	- Utilize both T1-weighted images and fiber orientation distribution function (fODF) as input	2D U-net (Ronneberger et al., 2015)	72	Edge enhancing diffusion filter	Dice: 0.832	N/A	N/A	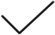
Wasserthal, Jakob, Peter Neher, and Klaus H. Maier-Hein/ 2018/ (Wasserthal et al., 2018)	HCP/105	- Utilizes fiber orientation distribution function (fODF) peaks as input - Semi-automatically generated binary segmentations for 72 tracts made public	2D U-net (Ronneberger et al., 2015) named TractSeg	72	Rotation, Elastic deformation, Displacement, Zooming, Resampling, Gaussian noise, Contrast augmentation, Brightness augmentation	Dice: 0.82 ~ 0.84	https://github.com/MIC-DKFZ/TractSeg/	1 min	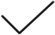
Ocegueda, Omar, and Mariano Rivera/2013/ (Ocegueda and Rivera, 2013)	2012 HARDI Reconstruction Challenge Dataset/− Phantom/−	- Represent DWI signal using a Muti-Tensor Field model - Points are embedded using eigenvectors to perform segmentation	Entropy-Controlled Quadratic Markov Measure Field (EC-QMMF)	16	N/A	Qualitative Results	N/A	14 min	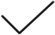

**Table 4:** Streamline-based clustering methods for Automated White Matter Tract Segmentation.

**Streamline-based clustering methods**								
**Author/Year/Citation**	**Dataset/No. of Subjects**	**Main Context**	**Clustering Algorithm**	**No. of fiber clusters**	**Performance Metrics**	**Practical Application**		
						**Code**	**Runtime** **per subject**	**External Validation**
Chen, Yuqian, et al./2023/ (Chen et al., 2023)	HCP/50; CNP/40; PPMI/30	- Trained using self-supervised learning with the pretext task of predicting pairwise fiber distances	K-Means; Deep Convolutional Embedded Clustering	800	Davies-Bouldin Index (DB): 2.014 ~ 2.119 White Matter Parcellation Generalization (WMPG): 0.970 ~ 0.996 Tract Anatomical Profile Coherence (TAPC): 0.830 ~ 0.844 Tract Surface Profile Coherence (TSPC): 0.476 ~ 0.601	https://github.com/SlicerDMRI/DFC	15 ~ 110 s	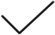
Zhao, Yi, et al./2022/ (Zhao et al., 2022)	HCP	- Multimodal dMRI and fMRI data (extracted BOLD signals) used as input for clustering	Riemannian metric geodesic distance to measure structural and functional differences for clustering fibers	72	Mean undirected euclidean distance (UE) Mean functional correlation (FC)	N/A	N/A	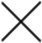
Chen, Yuqian, et al./2021/ (Chen et al., 2021)	HCP/200	- Based on self-supervised learning with the pretext task of pairwise fiber distance prediction	Siamese Networks, K-means, CNN	800	WMPG: 99.35% TAPC: 0.836	N/A	205 s	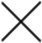
Xu, Chaoqing, et al./2021/ (Xu et al., 2021)	Private/−	- Based on encoding streamlines into 31 features and fed to encoder-decoder type architecture	Improved Deep Embedded Clustering (IDEC)	10	Qualitative results; Expert assessment	N/A	3 min	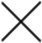
Logiraj, Kumaralingam, et al./2021/ (Logiraj et al., 2021a)	ADNI/20	- Based on geometrical curve features and multi-feature matching	Progressive clustering of large clusters of curves into smaller ones	6	Accuracy: 86% ~ 87%	N/A	N/A	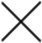
Vazquez, Andrea, et al./2020/ (Vázquez et al., 2020)	HARDI ARCHI/50	- Based on refining and merging clusters	Fast Fiber Clustering (FFClust)	150–200	Davies Bouldin Index (DB): 0.7 ~ 0.75 Execution Time: 1.99 min for 1 subject with 1 million fibers and parallel 45 s.	https://github.com/andvazva/FFClust	9.92 s	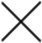
Yang, Zhipeng, et al./2020/ (Yang et al., 2020)	Private/7	- Based on using multi-modal information by combining spatial features and fMRI signals in WM	Gaussian Mixture Model (GMM) and Expectation Maximization (EM)	48	Hausdorff distance: 4.1 ~ 48.4	N/A	N/A	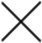
Siless, Viviana, et al./2018/ (Siless et al., 2018)	HCP/32	- Based on a novel anatomical similarity measure	Normalized Cuts (Brun et al., 2004; Shi and Malik, 2000)	200	Dice: 0.55–0.60	N/A	2.45 ~ 2392.37 min	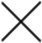
Gupta, Vikash, et al./2018/ (Gupta et al., 2018)	PPMI/226	- Use CNN to learn shape features and cluster streamlines	Convolutional Neural Network (CNN)	10	Accuracy: 97%	N/A	N/A	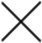
Gupta, Vikash, et al./2017/ (Gupta et al., 2017)	Private/42	- Use CNN to learn shape features and cluster streamlines	Convolutional Neural Network (CNN)	17	Qualitative results	N/A	N/A	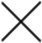
Román, Claudio, et al./2017/ (Roman et al., 2017)	Private/74	- Use intersubject hierarchical clustering of fibers - Create an atlas of identified bundles to promote automatic labeling	Heirarchical clustering	93	Lateralization index (Catani et al., 2012): –0.171 ~ 0.389	N/A	2.6 ~ 3.4 h	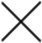
Kamali, Tahereh, and Daniel Stashuk/2016/ (Kamali and Stashuk, 2016)	JHU DTI (http://lbam.med.jhmi.edu)/15	- Based on distances of nearest neighbors of individual fibers - separate high densities (smaller distances) from lower densities (higher distances)	Neighborhood Distance Entropy Consistency (NDEC)	3	Dice: 0.94 Density-Based Clustering Validation (DBCV): 0.71	N/A	2 min	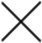
Kumar, Kuldeep, and Christian Desrosiers /2016/ (Kumar and Desrosiers, 2016)	HCP/10	- Atlas created from multi-subject data by learning a compact dictionary of training fibers describing the whole dataset	Kernel Sparse Clustering (KSC)	4	0.634 ~ 0.809	N/A	0.876 ~ 2.736 s	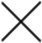
Jin, Yan, and H. Ertan Cetingül/2015/(Jin and Cetingül, 2015)	Neurospin MR phantom dataset (Poupon et al., 2008)/65 HCP/10	- Group fibers growing from a manually selected ROI and monitor divergence of fibers through drift detection while tractography is performed	Affinity Propagation (AP) (Frey and Dueck, 2007)	5	Dice Coefficient: 0.91 ~ 1.0	N/A	2 min	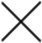
Tunç, Birkan, et al./2014/ (Tunc et al., 2014)	Private/6	- Based on a connectivity-based representation of fibers - Also generate a fiber clustering atlas which is used for further clustering unknown subjects	Gaussian Mixture Model (GMM) (Reynolds et al., 2000) and Expectation Maximization (EM) (Dempster et al., 1977)	327	Dice: 0.62 ~ 0.93	N/A	N/A	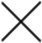

**Table 5:** Streamline-based classification methods for Automated White Matter Tract Segmentation.

**Streamline-based classification methods**								
**Author/Year/Citation**	**Dataset/No. of subjects**	**Main context**	**Architecture**	**No. of fiber clusters**	**Performance metrics**	**Practical application**		
						**Code**	**Runtime per subject**	**External validation**
Dumais, Félix, et al./2023/ (Dumais et al., 2023)	TractInferno (Poulin et al., 2022)/354; HCP/1200; MyeloInferno/45; ADNI/23; PPMI/34	- Based on training an autoencoder on contrastive loss using whole brain tractograms	AutoEncoder	27	Dice: 0.74 ± 0.08	https://github.com/scil-vital/fiesta	N/A	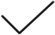
Bertò, Giulia, et al./2021/ (Bertò et al., 2021)	HCP-minor/105; HCP-IFOF/30; HCP-major/105; Private/10	- Based on vector representation using anatomical and geometrical information of streamlines	Linear Classifier	500	Dice: 0.80 ~ 0.91	https://brainlife.io	3 min	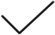
Logiraj, Kumaralingam, et al./2021/ (Logiraj et al., 2021b)	Private/15	- Based on segmenting 3D fiber curves into bundles	PointNet (Qi et al., 2017)	10	Accuracy: 97.06% Precision: 0.98 ~ 1.0 Recall: 0.91 ~ 1.0	N/A	N/A	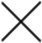
Zhang, Fan, et al./2020/ (Zhang et al., 2020)	HCP/100; dHCP/40; ABCD/50; CNP/50; PPMI/50; BTP/39	- Based on fiber descriptor called FiberMap (Zhang et al., 2020)	2D CNN	54	Accuracy: 90.99% Recall: 85.67% Precision: 88.47% Tract Identification Rate: 99.17% ~ 99.96% Weighted Dice: 0.91 ~ 0.97	http://dmri.slicer.org	8 min	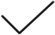
Wu, Ye, et al./2020/ (Wu et al., 2020)	HCP/105	- Based on representing each fiber bundle by compact dictionary	Dictionary Learning Tool DICTOL (Vu and Monga, 2017)	72	Accuracy: about 0.6–1.0	N/A	N/A	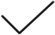
Zhang, Fan, et al./2019/(Zhang et al., 2019)	dHCP/40; ABIDE/70; HCP/100; CNP/204; PPI/144; BTP/39	- Based on fiber descriptor called FiberMap (Zhang et al., 2020)	2D CNN	54	Accuracy: 90.99% Recall: 85.67% Precision: 88.47% Tract Identification Rate: 99.17–100%	https://github.com/SlicerDMRI/DeepWMA	8 min	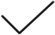
Liu, Feihong, et al./2019/ (Liu et al., 2019)	HCP/38	- Based on representing streamlines as graphs - Separate network trained for each bundle	Graph Convolutional Neural Network (GCNN)	12	Precision: 90.5% ~ 9.9% Recall: 88.4% ~ 100% Dice: 80.7 ~ 99.1	N/A	N/A	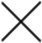
Ugurlu, Devran, et al./2019/(Ugurlu et al., 2019)	HCP/30	- Based on representing each streamline as the fiber orientation distributions in its neighborhood	NN	9	Bundle-based Minimum Distance (BMD): 1.2 ~ 5.46 Kappa: 0.68 ~ 0.84	N/A	N/A	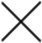
Bertò, Giulia, et al. /2019/ (Bertò et al., 2019)	HCP/130	- example created based on 130 tractograms and using the Automated Fiber Quantification (Yeatman et al., 2012) algorithm	Linear assignment problem for segmentation and ROI-based distance matrix	12	Dice: 0.84 ~ 0.87	doi: 10.25663/brainlife.app.122	N/A	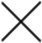
Lam, Prince D. Ngattai, et al./2018/ (Ngattai Lam et al., 2018)	Private/685	- Based on fiber features curvature, torsion and euclidean distances to a certain number of landmarks and CNN used to classify	2D NN	1	Accuracy: 98.8%	N/A	N/A	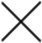
Heker, Michal, et al./2016/ (Heker et al., 2016)	HCP/15	- Based on Adaboost selected features such as fiber length, location, variance, etc.	Viola-Jones (Viola and Jones, 2001)	3	Dice: 0.90 ~ 0.91	N/A	N/A	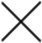
Ratnarajah, Nagulan, and Anqi Qiu/2014/ (Ratnarajah and Qiu, 2014)	GUSTO study (Soh et al., 2014)/20	- Based on Riemannian structure of diffusion tensors	Multi-label k-NN	13	Hamming Loss: 0.041 ~ 0.053 One error: 0.098 ~ 0.200 Coverage: 0.104 ~ 0.181 Volume Overlap percentage: 0.764	N/A	N/A	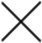

**Table 6:** Atlas-based methods for Automated White Matter Tract Segmentation.

**Atlas-Based Methods**								
**Author/Year/Citation**	**Dataset/No. of Subjects**	**Main Context**	**Architecture**	**No. of fiber clusters**	**Performance Metrics**	**Practical Application**		
						**Code**	**Runtime** **per subject**	**External Validation**
Radwan, ahmed M, et al./2022/ (Radwan et al., 2022)	HCP/20; MASSIVE/1	- Builds an atlas based on literature-based dissection protocol - atlas applied to new subjects using registration	ANTs (Avants et al., 2009)	68	Weighted-Dice: 0.747 ~ 0.963	https://github.com/KUL-Radneuron/KUL_FWT.git, https://osf.io/snq2d/	N/A	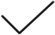
Jordan, Kesshi M., et al./2021/ (Jordan et al., 2021)	UCSF Dyslexia Center/59	- FreeSurfer derived ROIs used for anatomical information - RecoBundles (Garyfallidis et al., 2018) used to filter out the streamlines that do not match the shape of the tract based on predefined 3D bundle templates	Streamline Linear Registration	6	Dice: 0.76	https://github.com/kesshijordan/Kesh_Autoseg_Tools/tree/v1.0.0	N/A	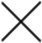
Vázquez, Andream et al./2019/ (Vázquez et al., 2019)	HARDI ARCHI/−	- Utilize Euclidean distance between subject fiber and atlas centroid using multi-subject bundle atlas	N/A	100/62 based on atlas used	Execution Time: 6 min	N/A	6 min	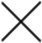
Zhang, Fan, et al./2018/ (Zhang et al., 2018)	HCP/200; dHCP/40; ABIDE/70; CNP/204; PPMI/144; BTP/26	- Atlas created based on data obtained across multiple populations and different scanners	Entropy-based tractography registration	256	White matter parcellation Generalization (WMPG): 92.28 ~ 100 Tract Anatomical Profile Coherence (TAPC): 0.626 ~ 0.783 Inter Subject Parcellation Variability (ISPV): 0.264 ~ 0.919	https://github.com/SlicerDMRI/whitematteranalysis	N/A	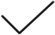
Sharmin, Nusrat, Emanuele Olivetti, and Paolo Avesani/2018/ (Sharmin et al., 2018)	HCP/30	- Based on finding corresponding streamlines across different tractograms formulated as a linear assignment problem (LAP)	FLIRT/FSL (Fischer and Modersitzki, 2003)	10	Dice: 0.40 ~ 0.80 Receiver Operating Characteristic (ROC): 0.75 ~ 0.90	https://github.com/FBK-NILab/LAP_tract_segmentation	2 min	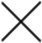
Labra, Nicole, et al./2017/ (Labra et al., 2017)	HARDI ARCHI/	- Compare subject streamlines to multisubject bundle atlas based on distance metric	N/A	26	Execution Time: 9 million streamlines in less than 6 min	integrated with the Brain VISA/Connectomist software (Duclap et al., 2012)	1 ~ 6.5 s	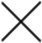
Yoo, Sang Wook, et al./2015/ (Yoo et al., 2015)	NMR/12	- Based on searching the most similar tract group in example data - multiple example subjects used; final label chosen based on voting scheme	FLIRT/FSL (Fischer and Modersitzki, 2003)	7	Consistency: 96.1% Sensitivity: 89.5% ~ 91.0% False Discovery Rate (FDR): 14.2% ~ 14.9% Kappa Analysis: 0.87 ~ 0.88	N/A	53.1 s	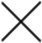
Jin, Yan, et al./2013/ (Jin et al., 2013)	HARDI/86	- Based on incorporating information from multiple hand-labeled atlases	ANTs (Avants et al., 2009)	17	Dice: 0.90 ~ 1.0	N/A	N/A	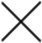

**Table 7:** Hybrid methods for Automated White Matter Tract Segmentation.

**Hybrid Methods**								
**Author/Year/Citation**	**Dataset/** **No. of subjects**	**Hybrid algorithms**		**No. of fiber bundles**	**Performance metrics**	**Practical application**		
						**Code**	**Runtime per subject**	**External validation**
Xu, H., et al./2023/ (Xu et al., 2023)	HCP/105	Registration Deep learning-based registration (Balakrishnan et al., 2019)	Segmentation TractSeg (Wasserthal et al., 2018)	72	Dice: 73.01%	https://github.com/HaoXu0507/ISBI2023-One-Shot-WM-Tract-Segmentation	N/A	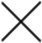
Peretzke, Robin, et al./2023/ (Peretzke et al., 2023)	HCP/21 Private/10	Semi-Automatic Based on an active learning pipeline by training a random forest classifier on a specific tract	Manual Unlabeled streamlines from whole brain tractogram are manually annotated	3	Dice: 0.73 ~ 0.90	https://github.com/MIC-DKFZ/MITK-Diffusion	N/A	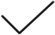
Delmonte, Alessandro, et al./2019/ (Delmonte et al., 2019)	HCP/5	Semi-Automatic Representing the inherent inaccuracy of anatomical definitions using theory of fuzzy sets (Bloch, 2005)	Manual Model qualitative anatomical definitions, navigate through levels of resolution	2	Qualitative Results	https://github.com/CorentinMercier/FBTS	100 s	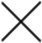
Garyfallidis, Eleftherios, et al./2018/ (Garyfallidis et al., 2018)	BIL&GIN diffusion data (Mazoyer et al., 2016)/60	Clustering Quickbundles (Garyfallidis et al., 2012)	Registration Streamline-based Linear Registration (SLR)	4	Jaccard index: 0.21 ~ 0.26 Accuracy: 0.99 ~ 1.0 Sensitivity: 0.68 ~ 0.92 Specificity: 1.0 Bundle Adjacency: 0.53 ~ 0.68	http://dipy.org	N/A	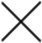
O’Donell, Lauren J./2017/ (O’Donnell et al., 2017)	HCP/10; Private/18	Atlas-based Atlas learned using groupwise-registration and spectral clustering	Registration tractography-based registration to atlas	800	Accuracy: 80% ~ 94%	https://github.com/SlicerDMRI/whitematteranalysis	2.5 h	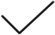
Wassermann, Demian, et al. /2016/ (Wassermann et al., 2016)	Private/77	Semi-Automatic: - a novel query language based on a near to English textual syntax to construct a dictionary of anatomical definitions describing white matter tracts	Manual: - tract descriptions are written by the operator as text sentences	32	Kappa score: 0.71 ~ 0.90	https://demianw.github.com/tract_querier	N/A	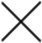
Chekir, Amira, et al./2014/ (Chekir et al., 2014)	HARDI/3;	Clustering: Quickbundles (Garyfallidis et al., 2012)	Atlas-based: WMPM Type 2 Eve Atlas	13	Kappa analysis: 0.70 Quantitative Diffusivity Analysis (FA average correlation): 0.94	N/A	N/A	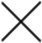
Wassermann, Demian, et al./2013/ (Wassermann et al., 2013)	Private/77	Semi-Automatic: - a novel query language based on a near to English textual syntax to construct a dictionary of anatomical definitions describing white matter tracts	Manual - careful syntactical definition of major white matter tracts in the human brain based on a neuroanatomist’s expert knowledge	37	Mean FA to detect tract changes specific to schizophrenia	https://demianw.github.com/tract_querier	N/A	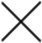

#### 3.2.1 Direct voxel-based methods

**Figure 3:**
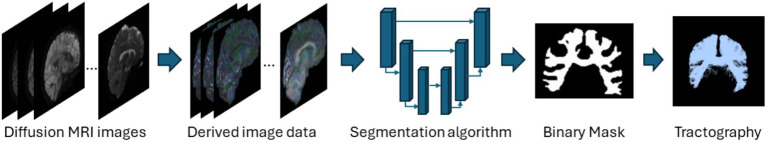
Illustration of the direct voxel-based segmentation pipeline using the segmentation of the corpus callosum as a representative example. Refer to Table 3 for more details regarding the direct voxel-based segmentation methods.

This category of methods directly segments tracts based on the diffusion images without performing tractography as shown in Figure 3. These methods are fast and utilize deep learning or machine learning techniques like convolutional neural networks (CNNs) to improve segmentation accuracy. Direct segmentation helps in providing a simpler processing pipeline and reduces potential errors due intermediate steps like registration (Mancini et al., 2019). Voxel-based approaches can associate each voxel with multiple tracts which is useful since WM tracts are known to cross or overlap (Jeurissen et al., 2019). Recent advances in GPU-based algorithms reduce algorithm runtimes to several minutes due to their highly parallelizable implementations. Although learning-based techniques achieve very high segmentation performance and are fast, they require a large number of manually annotated training data. Manual annotations are labor intensive to obtain, time-consuming and are prone to inter-observer intra-observer or even inter training set variability. Deep learning models also fail to generalize well on unseen data if they are trained on scarce training scans. Table 3 provides a list of all studies that use direct voxel-based approaches.

#### 3.2.2 Streamline-based clustering

**Figure 4:**
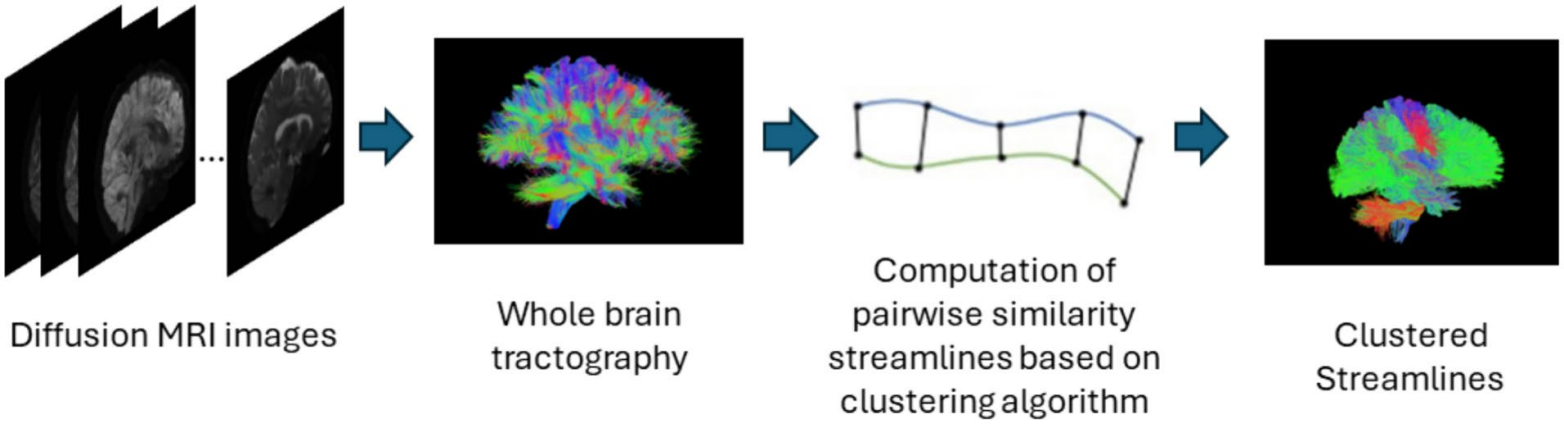
Illustration of the streamline-based clustering pipeline. Refer to Table 4 for more details regarding the clustering methods included in this review.

Streamline based methods are those that are applied to streamlines derived from whole brain tractography outputs as shown in Figure 4. These streamlines can be clustered or classified into meaningful groups of fibers known as bundles in either supervised or unsupervised ways. The unsupervised approach usually called streamline clustering methods are a popular white matter tract segmentation method. Such methods divide the entire brain white matter into multiple white matter parcels based on some information about the streamlines. Several bundles can be found using clustering-based methods, and the tractography data can also be characterized by using these clusters and their centroids as representative data which is used for further analyses. One of the main steps after clustering is to assign a label to the clustering results. This is a crucial step since clustering methods are commonly criticized to provide no guarantee of obtaining anatomically meaningful tracts (Toga and Mazziotta, 2002). Therefore in many cases, prior knowledge is used for this purpose, for example, by using an ROI atlas to guide the identification (Logiraj et al., 2021a) or from labeling clusters of streamlines from multiple subjects also called as atlas creation (Yoo et al., 2015; Labra et al., 2017) or labeling clusters in a single subject (Garyfallidis et al., 2018). Recently, deep learning methods are also being used for clustering large tractography datasets (Zhang et al., 2020). One of the main limitations of such methods is the large size of tractography datasets which are composed of various tracts of different shapes, lengths, positions. The advent of improved dMRI techniques has resulted in increased size and complexity of datasets. Tractography datasets comprise up to more than 10 million tracts. This causes an increase in storage and memory challenges when clustering such large datasets. Table 4 provides a list of all studies that use streamline-based clustering approaches.

#### 3.2.3 Streamline-based classification

**Figure 5:**
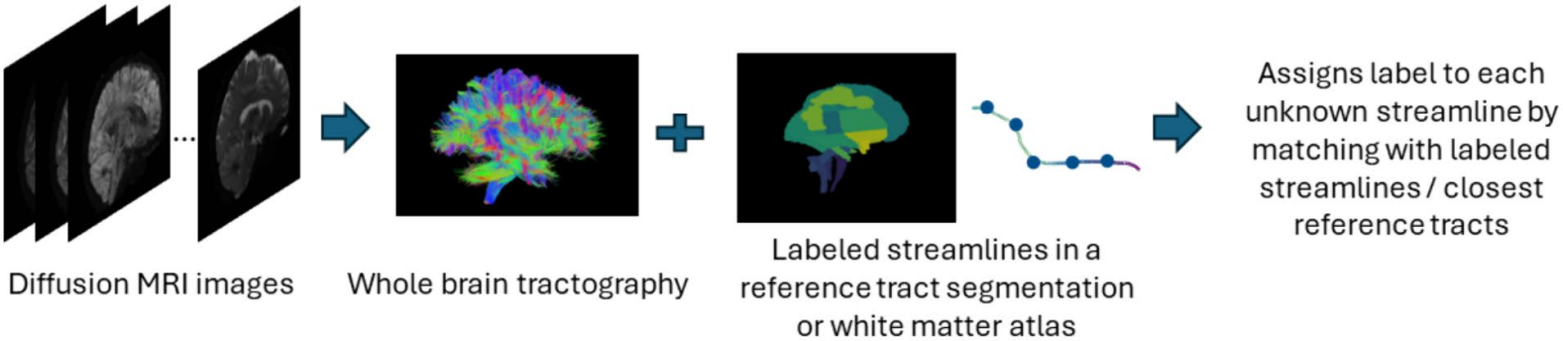
Illustration of the streamline-based classification pipeline. Refer to Table 5 for more details regarding the classification methods included in this review used to assign labels to streamlines.

The supervised approach of streamline-based methods involves streamline-based classification or labeling as shown in Figure 5. These methods assign an anatomical label to each individual streamline. This can be done by computing a pairwise distance of each streamline to a labeled streamline in a reference tract segmentation and then assigning a streamline label based on the closest reference tract (Bertò et al., 2021). Recently, fibers obtained after tractography are classified into tracts using a deep learning-based classifiers such as CNNs which are trained on selected fiber features. Similar to segmentation methods, while they are fast in assigning labels to fibers, they also require a large number of manually annotated training data and tend to face similar issues as segmentation methods. Table 5 provides a list of all studies that use streamline-based classification approaches for automated methods for white matter tract segmentation.

#### 3.2.4 Atlas-based

**Figure 6:**
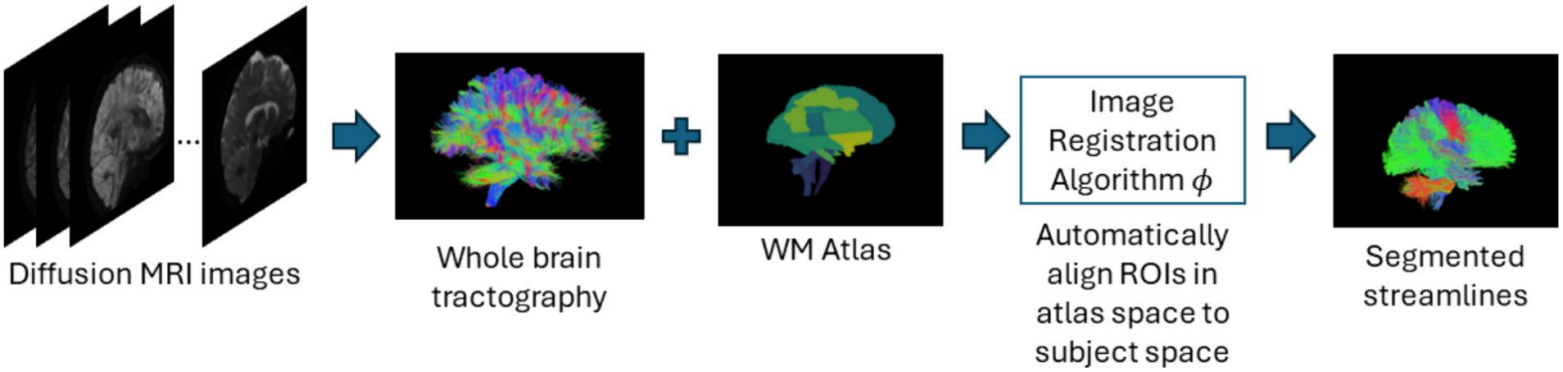
Illustration of the atlas-based method pipeline. Refer to Table 6 for more details regarding the atlas-based methods included in this review.

In atlas-based methods, tracts are identified by automatic placement of ROIs by warping a brain ROI atlas (Cook et al., 2005) or using volumes of interest (Oishi et al., 2009) to automatically group fiber streamlines into anatomically defined tracts as shown in Figure 6. These methods also can be based on tract similarity, also called streamline-based methods, using pairwise tract distances with a reference streamline label and assign a label based on the reference label of the streamline it is closest to (O'Donnell and Westin, 2007; Wu et al., 2020). Such approaches require image-based multi-modal nonlinear registration so that the streamlines obtained from tractography, and the ROIs are in the same space. However, registration results are not perfect because aligning streamlines with ROIs is a challenging task and time-consuming and can be even more difficult when applied to pathological brains. While more tracts can be easily added to the reference streamline atlas, in such methods, limited quality of some tracts limits their generalization ability. Table 6 provides a list of all studies that use atlas-based approaches.

#### 3.2.5 Hybrid

In this review we also identified methods that combined more than one strategy from the categories to extract more information to improve labeling of anatomical bundles. The semi-automated methods identified in this review are also included under this category. Semi-automated techniques typically involve human intervention, such as manual labeling or correction, within an otherwise automated process. These methods are more time consuming since they have multiple steps as compared to the other methods. Table 7 provides a list of all studies that use hybrid approaches.

### 3.3 Evaluation metrics

In this section we review the most common evaluation methods that have been used for validating the white matter tract segmentation results in the studies included in this work. Evaluation of accuracy for tract segmentation is difficult since the errors cannot point out which stage of the pipeline causes the issue; for example, it is difficult to determine whether the errors were generated from the preprocessing steps, the selected algorithm for tract segmentation, similarity metric, etc. Table 8 gives a list of the most frequently used evaluation metrics with the following attributes for each: the metric name, metric description provides a brief definition, the formulation of the metric to show how it is computed and finally the usage of the metric.

**Table 8:** Evaluation metrics used for validating Automated White Matter Tract Segmentation.

**Evaluation Metrics**			
**Metric**	**Description**	**Formulation**	**Usage**
Dice Coefficient (DSC)	Calculate overlap between segmented tract and the groundtruth tract. Convert tracts into binary masks where 1 indicates that a voxel is crossed by a streamline of the tract and 0 otherwise.	Given the segmented tract $t^{\prime }$, and the ground truth tract t, $DSC=\frac{2\times \left(\left|v\left(t^{\prime }\right)\cap v\left(t\right)\right|\right)\;}{\left(\left|v\left(t^{\prime }\right)+v\left(t\right)\right|\right)}$	The authors in Zhang et al. (2018) proposed Tract Anatomical Profile Coherence (TAPC) metric and Tract Surface Profile Coherence (TSPC) which are both based on the Dice scores computed between either for the tract anatomical profile or tract surface profile.
Jaccard Similarity Index (JSI)	Measure the ratio of the intersection of voxels belonging to a predicted tract with its groundtruth and the union of all voxels belonging to a predicted tract and its groundtruth.	For a predicted segmented tract $t^{\prime }$, and the ground truth tract t, $JI=\;\frac{|v\left(t^{\prime }\right)\cap v\left(t\right)|\;}{\left|v\left(t^{\prime }\right)\cup v\left(t\right)\right|}$ where $v\left(t\right)$ is the set of voxels crossed by the streamlines of t, and $v\left(t^{\prime }\right)$ us the set of voxels crossed by streamlines of $t^{\prime }$.	The score ranges from 0 to 1, with 1 showing exact similarity and 0 showing no similarity between two tract segments. This is also referred to as the volumetric overlap error (VOE) in segmentation methods (Chen et al., 2012).
Precision	Normalize the volume of the correctly segmented tract over the volume of the result of the segmentation.	For a predicted segmented tract $t^{\prime }$, and the ground truth tract t, $\mathrm{Precision}=\frac{\left(\left|v\left(t^{\prime }\right)\cap v\left(t\right)\right|\right)\;}{v\left(t^{\prime }\right)}$	Precision ranging from 0 to 1 focuses on the proportion of positive predictions that were correct.
Recall	Normalize the size of the correctly segmented tract over the ground truth tract segmentation	For a predicted segmented tract $t^{\prime }$, and the ground truth tract t, $\mathrm{Recall}=\frac{\left(\left|v\left(t^{\prime }\right)\cap v\left(t\right)\right|\right)\;}{v\left(t\right)}$	Recall ranging from 0 to 1 focuses on the proportion of actual positive instances that were correctly identified.
Davies-Bouldin Index (DB)	Measure the average similarity of each cluster with its most similar cluster, where similarity is the ratio of within-cluster distances to between-cluster distances (Davies and Bouldin, 1979)	$DB=\frac{1}{k}\overset{\dot{i}=1}{\underset{k}{\sum }}\max_{i\neq j}\;R_{ij}$ where k is the number of clusters, $R_{ij}$ is the ratio of the average within-cluster distance to the between-cluster distance.	Evaluates the white matter tract segmentation methods that are based on clustering approaches. A lower DB score shows better clustering results, with 0 being the minimum score.
Density Based Clustering Validation (DBCV) (Moulavi et al., 2014)	Assigns a validity index to the obtained clustering solution which considers both the density and shape properties of the clusters.	$\mathrm{DBCV}\left(C\right)=\overset{i=l}{\underset{i^{1}=1}{\sum }}\frac{\left|C_{i}\right|}{\left|O\right|}V_{C}\left(C_{i}\right)$ where a validity index of a clustering solution is obtained by taking the weighted average of the validity indexes for all clusters given a**s** $V_{C}\left(C_{i}\right)$, l are the number of clusters, $\left|O\right|$ is the number of objects in the cluster.	This results in a score between [−1, 1], with greater values indicating better clustering solutions.
Kappa analysis	Evaluate agreement between two raters, which is known to be robust since the kappa considers agreement by chance (Lacante et al., 2008)	Two binary images are superimposed to classify each pixel into three categories: pixels whose values are 1 in both images (pp), pixels whose values are 0 in both images (nn), and pixels whose values are different in the two images (pn, np). Then a probability of observed agreement ($p_{0}$) and a probability of chance agreement ($p_{e}$) are computed as follows: $p_{0}=\frac{\mathrm{pp}+nn}{N}$ $p_{e}=\left(\frac{\mathrm{pp}+pn}{N}\right).\left(\frac{\mathrm{pp}+np}{N}\right)+\left(\frac{nn+np}{N}\right).\left(\frac{nn+pn}{N}\right)$ where N=pp+nn+pn+np.Finally the kappa value, κ, for the two bundles is computed as follows: $\kappa =\frac{p_{0}-p_{e}}{1-p_{e}}$	Landis and Koch assigned labels to kappa value ranges as follows [103]: κ value smaller than 0 is “poor,” 0.00–0.20 is “slight,” 0.21–0.40 is “fair,” 0.41–0.60 is “moderate,” 0.61–0.80 is “substantial” and 0.81–1.00 is “almost perfect” agreement. For each bundle, a binary image is computed which is the same size as the diffusion-weighted image, by setting pixel value to 1 if any tract passes through the voxel and set to 0 otherwise.

Other than these commonly used evaluation methods, white matter tract segmentation methods are also validated qualitatively in the form of visualizing the generated tract segmentation. Visualization by a domain expert is still used as a complementary method along with a few of the above-mentioned quantitative measures. Recently authors in Pujol et al. (2015) had initiated the DTI challenge to promote the standardized evaluation of tractography methods for neurosurgery. Despite ample research in the development of tractography and tract segmentation algorithms there is no consensus on the validation techniques to compare the different algorithms.

## 4 Discussion

In this review paper, we have provided a systematic review of automated methods for white matter tract segmentation with respect to the most widely used public datasets for this task, the various categories of automated methods developed, and the evaluation metrics used to study the performance of the method. Although there are studies that have reviewed automated methods for brain tractography (Poulin et al., 2019; Zhang et al., 2022) and also deep learning methods for tract segmentation (Ghazi et al., 2023), to the best of our knowledge, a systematic review that focuses on automated methods for tract segmentation has not been published yet. This review paper underscores the methodological advancements in building automated methods, as evidenced through the 59 articles included in this review.

While manual segmentation of tracts or virtual dissection methods were not a focus of this survey, multiple approaches have been proposed in the last decade that conduct fiber selection and anatomical labeling using expert knowledge (Rheault et al., 2020, 2022a,b; Ille et al., 2021). These methods focus on improving the design of white matter dissection protocols to build more generalizable and reproducible methods. In Schilling et al. (2021) authors show the need to have a standard nomenclature and definitions for white matter bundles and that there are still issues in tractography segmentation that need to be resolved so that they can be used in routine clinical settings. Such methods are worth mentioning in this survey since they show that segmenting white matter tracts is a crucial task and that there is still a lot of scope for improvement.

It is also important to note that our study of white matter tract segmentation focusses on fibers in the deep white matter. Multiple studies are available that investigate the segmentation of subcortical U-fibers, which are special types of short association fibers located in the superficial white matter (Guevara et al., 2017, 2020; Xue et al., 2023). Despite studies on superficial white matter (SWM) being sparse due to its complexity (Xue et al., 2023) have employed point-cloud-based deep learning techniques that concentrate on superficial white matter tract segmentation. Also, numerous studies on automated white matter tract segmentation methods were omitted from this review because they did not meet the search criteria used to compile the literature included in this study. For example, studies (Bazin et al., 2011; Yendiki et al., 2011) provide automated methods for white matter tract segmentation, however, were not included in this study since they are published before 2013. Studies centered on automated fiber tracking or tractography (Teeuw et al., 2015; Warrington et al., 2020) are not featured in this review; however, they merit attention as they play a vital role in advancing new automated methodologies for accurately reconstructing white matter pathways, thereby facilitating the analysis of extensive datasets. Authors in Warrington et al. (2020) present tractography protocols as a software tool for standardized and automated cross-species tractography generated from large datasets. Automated tractography methods such as TRACULA (Yendiki et al., 2011), Teeuw et al. (2015) have used learning-based methods that show highly promising performance incorporating information on the anatomy of the pathways for reconstruction of white matter pathways thereby facilitating automated fiber tracking to large studies.

Twenty-seven percent of the papers in the current survey are based on direct voxel-based segmentation methods. Our results show that papers based on fully convolutional networks are typically based on encoder-decoder architecture such as U-net (Ronneberger et al., 2015). These voxel-based methods are gaining popularity with the advent of new and efficient deep learning-based segmentation techniques. However most current studies still rely on U-net based architectures as the baseline model, and the popular segmentation architectures like those based on transformers (Dosovitskiy et al., 2020; Hatamizadeh et al., 2021; Cao et al., 2022) have not been applied to this domain yet. Deep learning methods also perform segmentation by either labeling the streamlines or directly labeling the voxels. In general, the progress seen in using deep learning methods for medical image segmentation tasks (Hatamizadeh et al., 2021; Xiao et al., 2023) has not yet been fully applied to white matter tract segmentation. This is mostly because it is more demanding to have manual annotations of white matter tracts than other brain anatomical structures. Also, while deep learning methods provide fast segmentations, their results can still be unsatisfactory, and are not robust to changes of bundle sizes, tracking methods and data quality (Bertò et al., 2021). This shows that the major challenges in using machine learning or deep learning methods for tract segmentation will require researchers to come up with more generalizable solutions, create and publish more annotated datasets, use other techniques like transfer learning, self-supervised learning to overcome the challenges of limited training samples for deep learning-based methods to gain clinical applicability.

Next, our results show 25% of the papers in the current survey applied streamline-based clustering methods and 20% used streamline-based classification methods for automatic tract segmentation. These methods focus on clustering large number of fiber trajectories or streamlines into clusters or fiber bundles. However, few of these methods attach labels to clusters, and the clusters must be assigned labels either manually or automatically by using a streamline atlas usually incorporated in the clustering process. Such methods also have to post-process their results in order to filter the bundles to exclude spurious tracts that are falsely included in the clustering results.

In all the methods seen in this work, only two studies were found which used registration-based methods for white matter tract segmentation (Garyfallidis et al., 2018; Jordan et al., 2021). In Wasserthal et al. (2018) authors compared their work with two registration-based methods for automatic tract segmentation, which usually involves using a tract atlas and registering it to the subject of interest which yields a binary mask for each tract in subject space. Fewer registration methods are likely employed due to the inaccuracies produced during the registration step, and the computational complexity needed. However recently, a lot of work has been done in using deep learning methods for image registration (Oliveira and Tavares, 2014; Fu et al., 2020) to overcome the challenges of traditional registration methods. For example, authors in Zhang et al. (2021) proposed a deep learning-based method for registration of dMRI images. This exemplifies the growing interest in applying such methods to tract segmentation. Currently, to the best of our knowledge, there are no studies that use deep learning-based registration techniques for the task of automated white matter tract segmentation.

It is interesting to note that there is limited existing work on automated methods for segmenting white matter bundles for the neonatal brain. In our survey we only found two papers (Ratnarajah and Qiu, 2014; Logiraj et al., 2021b) that correspond to this topic. This could mainly be because segmenting white matter structures is particularly difficult in the neonatal brain since it is undergoing a critical growing process along with cellular maturation such as myelination and synaptic pruning (Ratnarajah and Qiu, 2014). Existing methods rely mostly on fully manual segmentation for delineating white matter structures (Oishi et al., 2011) or are based on semi-automated techniques (Huang et al., 2006). In Oishi et al. (2011) authors developed an atlas-based segmentation based on image registration, which also needs manual expert assessment in order to delineate the required white matter structures. This work was developed almost a decade ago and there have been multiple automated segmentation techniques proposed since that have been successfully applied to adult’s brain as shown in this survey. Manual methods also suffer challenges of being time consuming and require prior anatomical knowledge to achieve reasonable accuracy and reproducibility.

Overall, we observe that automated tract segmentation algorithms follow varied methods for pre-processing, augmenting, and training their datasets and few methods use multi-site datasets. Even the techniques used to generate reference tracts are not the same across most of the methods. This makes it impossible to assess the true generalizability and reliability of the proposed methods (Poulin et al., 2022). This problem is also observed in manual segmentation methods where there is varied reproducibility for segmenting the same tracts among different experts or the inter-protocol agreement across protocols for various white matter pathways is poor as shown in (Schilling et al., 2021).

We observe that most studies included in this review do not provide computational time making it challenging to assess the practicality of these methods. In general, streamline-based methods typically require substantial memory for generating millions of streamlines per subject, whereas direct voxel-based methods can segment white matter tracts for a test subject in under a minute (Wasserthal et al., 2018). Out of 59 segmentation methods reviewed, only 18 have been validated on external datasets with varying scanners and acquisition parameters. This lack of generalizability testing may be due to the limited availability of publicly accessible tract segmentation datasets. Despite this, direct voxel-based methods can be used for data augmentation during training to simulate domain shifts in external datasets, potentially reducing the domain-shift impact.

Lastly, we have summarized the most common evaluation metrics used by tract segmentation methods to validate their results in Section 3.3. However, there is no consensus on the evaluation metrics used to compare the various proposed approaches. Due to the limitation of ground truth, most methods rely on reproducibility in terms of intra- and inter-rater as well as test–retest reproducibility (Zhang et al., 2019; Rheault et al., 2020, 2022b) and consistency of methods across different populations and acquisitions (Wasserthal et al., 2018, 2019), as validations points for identifying a good tract segmentation method.

## 5 Future directions

Although this paper reveals the advancements of automated methods for white matter tract segmentation, there is still a lack of a general standardized method that can be reliably used by clinicians. There is still limited consensus on the definition of tracts even among knowledgeable and experienced professionals who are concerned about the inter- and intra-user reproducibility with manual placement of ROIs (Zhang et al., 2010). This further complicates the methodology development and validation process. This suggests that there is a need for the development of more standardized approaches for validation tract segmentation results.

The recent work of authors of TractSeg (Wasserthal et al., 2018) enabled the distribution of manually labelled tracts to the community so that researchers could collaboratively share the segmented tracts by experts. This gave rise to the development of more generalized approaches towards white matter tract segmentation, which otherwise would not have been possible. This has set a particularly good example so that in the future, researchers can continue to enable the progress, development, and assessment of higher-quality automated methods through such public collaborations. There is still substantial room for future improvements in the domain of generating high quality ground truth via expert neuroanatomists.

Another important aspect to consider when developing automated methods is their computational cost. With the advent of improved imaging tools for the acquisition of data and increasing efficiency of computational resources, there is a critical need for building applications that can be clinically used. Moreover, there has been a significant surge in image sizes. A decade ago, state-of-the-art MR acquisitions typically featured MR images of human brains with voxel sizes of 2 × 2 × 2 mm^3^. Today, we routinely encounter voxel sizes smaller than 1 × 1 × 1 mm^3^, as seen in data collected by projects like the Human Connectome Project (Van Essen et al., 2012; Glasser et al., 2016). Therefore, rapid tract segmentation approaches are needed to allow interactive analysis and also to efficiently handle very large imaging studies in a time and cost-effective manner.

Another important future direction would be to consider tractogram data generated from varied tracking algorithms as input to the automated tract segmentation methods developed. This is because a variety of tracking algorithms with different parameter values can be used by tractography studies to generate the tractograms. Then the segmentation of tracts could be applied to any of these generated tractograms, and the method should be able to adapt to all these diverse types of inputs.

## 6 Conclusion

This systematic review summarized 59 relevant articles in all. Unlike previous studies, our work focuses on a systematic review of methods for automated white matter tract segmentation developed in the last decade. This work framed crucial research questions to explain what approaches have been used for automated tract segmentation methods, discover key research gaps, determine datasets that are publicly accessible for researchers and summarize the most common evaluation techniques utilized. The literature published in this area as displayed and characterized in the Results section is one that is of growing and global interest.

## Data availability statement

The original contributions presented in the study are included in the article/supplementary material, further inquiries can be directed to the corresponding author.

## Author contributions

AJ: Conceptualization, Data curation, Formal analysis, Investigation, Methodology, Software, Validation, Visualization, Writing – original draft, Writing – review & editing. HL: Investigation, Project administration, Resources, Supervision, Writing – review & editing. NP: Funding acquisition, Project administration, Resources, Supervision, Writing – review & editing. LH: Funding acquisition, Project administration, Resources, Supervision, Writing – review & editing.

## References

[ref1] Avants B. B. Tustison N. Song G. (2009). Advanced normalization tools (ANTS). Insight J. 2, 1–35.

[ref2] Balakrishnan G. Zhao A. Sabuncu M. R. Guttag J. Dalca A. V. (2019). VoxelMorph: a learning framework for deformable medical image registration. IEEE Trans. Med. Imaging 38, 1788–1800. doi: 10.1109/TMI.2019.2897538, PMID: 30716034

[ref3] Basser P. J. Mattiello J. LeBihan D. (1994a). Estimation of the effective self-diffusion tensor from the NMR spin echo. J. Magn. Reson. B 103, 247–254. doi: 10.1006/jmrb.1994.1037, PMID: 8019776

[ref4] Basser P. J. Mattiello J. LeBihan D. (1994b). MR diffusion tensor spectroscopy and imaging. Biophys. J. 66, 259–267. doi: 10.1016/S0006-3495(94)80775-1, PMID: 8130344 PMC1275686

[ref5] Basser P. J. Pajevic S. Pierpaoli C. Duda J. Aldroubi A. (2000). In vivo fiber tractography using DT-MRI data. Magn. Reson. Med. 44, 625–632. doi: 10.1002/1522-2594(200010)44:4<625::AID-MRM17>3.0.CO;2-O, PMID: 11025519

[ref6] Bazin P. L. Ye C. Bogovic J. A. Shiee N. Reich D. S. Prince J. L. . (2011). Direct segmentation of the major white matter tracts in diffusion tensor images. NeuroImage 58, 458–468. doi: 10.1016/j.neuroimage.2011.06.020, PMID: 21718790 PMC3159825

[ref7] Bertò G. Avesani P. Pestilli F. Bullock D. Caron B. Olivetti E. (2019). Anatomically-informed multiple linear assignment problems for white matter bundle segmentation. 2019 IEEE 16th International Symposium on Biomedical Imaging (ISBI 2019),

[ref8] Bertò G. Bullock D. Astolfi P. Hayashi S. Zigiotto L. Annicchiarico L. . (2021). Classifyber, a robust streamline-based linear classifier for white matter bundle segmentation. NeuroImage 224:117402. doi: 10.1016/j.neuroimage.2020.117402, PMID: 32979520

[ref9] Bloch I. (2005). Fuzzy spatial relationships for image processing and interpretation: a review. Image Vis. Comput. 23, 89–110. doi: 10.1016/j.imavis.2004.06.013

[ref10] Brun A. Knutsson H. Park H.-J. Shenton M. E. Westin C.-F. (2004). Clustering fiber traces using normalized cuts. Medical Image Computing and Computer-Assisted Intervention–MICCAI 2004: 7th International Conference, Saint-Malo, France, September 26–29, 2004. Proceedings, Part I 7 10.1007/b100265 PMC3296487 20209048

[ref11] Bullock D. Takemura H. Caiafa C. F. Kitchell L. McPherson B. Caron B. . (2019). Associative white matter connecting the dorsal and ventral posterior human cortex. Brain Struct. Funct. 224, 2631–2660. doi: 10.1007/s00429-019-01907-8, PMID: 31342157

[ref12] Cao H. Wang Y. Chen J. Jiang D. Zhang X. Tian Q. . (2022). Swin-unet: Unet-like pure transformer for medical image segmentation. European conference on computer vision

[ref13] Carter M. Jennifer S. Farra N. Harris G. ScienceDirect (2015). Guide to research techniques in neuroscience. 2nd Edn Academic Press.

[ref14] Catani M. (2006). Diffusion tensor magnetic resonance imaging tractography in cognitive disorders. Curr. Opin. Neurol. 19, 599–606. doi: 10.1097/01.wco.0000247610.44106.3f 17102700

[ref15] Catani M. Dell'acqua F. Vergani F. Malik F. Hodge H. Roy P. . (2012). Short frontal lobe connections of the human brain. Cortex 48, 273–291. doi: 10.1016/j.cortex.2011.12.001 22209688

[ref16] Catani M. Howard R. J. Pajevic S. Jones D. K. (2002). Virtual in vivo interactive dissection of white matter fasciculi in the human brain. NeuroImage 17, 77–94. doi: 10.1006/nimg.2002.1136 12482069

[ref17] Chekir A. Descoteaux M. Garyfallidis E. Côté M.-A. Boumghar F. O. (2014). A hybrid approach for optimal automatic segmentation of white matter tracts in hardi. 2014 IEEE Conference on Biomedical Engineering and Sciences (IECBES)

[ref18] Chen Z. Tie Y. Olubiyi O. Zhang F. Mehrtash A. Rigolo L. . (2016). Corticospinal tract modeling for neurosurgical planning by tracking through regions of peritumoral edema and crossing fibers using two-tensor unscented Kalman filter tractography. Int. J. Comput. Assist. Radiol. Surg. 11, 1475–1486. doi: 10.1007/s11548-015-1344-5, PMID: 26762104 PMC4942409

[ref19] Chen X. Udupa J. K. Bagci U. Zhuge Y. Yao J. (2012). Medical image segmentation by combining graph cuts and oriented active appearance models. IEEE Trans. Image Process. 21, 2035–2046. doi: 10.1109/TIP.2012.2186306, PMID: 22311862 PMC5548181

[ref20] Chen Y. Zhang C. Song Y. Makris N. Rathi Y. Cai W. . (2021). Deep fiber clustering: anatomically informed unsupervised deep learning for fast and effective white matter parcellation. International Conference on Medical Image Computing and Computer-Assisted Intervention

[ref21] Chen Y. Zhang C. Xue T. Song Y. Makris N. Rathi Y. . (2023). Deep fiber clustering: anatomically informed fiber clustering with self-supervised deep learning for fast and effective tractography parcellation. NeuroImage 273:120086. doi: 10.1016/j.neuroimage.2023.120086, PMID: 37019346 PMC10958986

[ref22] Clayden J. D. Storkey A. J. Bastin M. E. (2007). A probabilistic model-based approach to consistent white matter tract segmentation. IEEE Trans. Med. Imaging 26, 1555–1561. doi: 10.1109/TMI.2007.905826, PMID: 18041270

[ref23] Cook P. A. Zhang H. Avants B. B. Yushkevich P. Alexander D. C. Gee J. C. .. (2005). An automated approach to connectivity-based partitioning of brain structures. Medical image computing and computer-assisted intervention–MICCAI 2005: 8th International Conference, Palm Springs, CA, USA, October 26–29, 2005, Proceedings, Part I 8 10.1007/11566465_21 16685842

[ref24] Davies D. L. Bouldin D. W. (1979). A cluster separation measure. IEEE Transac. Pattern Analysis Machine PAMI-1, 224–227. doi: 10.1109/TPAMI.1979.4766909 21868852

[ref25] De Belder F. E. Oot A. R. Van Hecke W. Venstermans C. Menovsky T. Van Marck V. . (2012). Diffusion tensor imaging provides an insight into the microstructure of meningiomas, high-grade gliomas, and peritumoral edema. J. Comput. Assist. Tomogr. 36, 577–582. doi: 10.1097/RCT.0b013e318261e913, PMID: 22992609

[ref26] Delmarcelle T. Hesselink L. (1992). Visualization of second order tensor fields and matrix data. In Proceedings Visualization’92. (pp. 316–317). IEEE Computer Society.

[ref27] Delmonte A. Mercier C. Pallud J. Bloch I. Gori P. (2019). White matter multi-resolution segmentation using fuzzy set theory. 2019 IEEE 16th International Symposium on Biomedical Imaging (ISBI 2019)

[ref28] Dempster A. P. Laird N. M. Rubin D. B. (1977). Maximum likelihood from incomplete data via the EM algorithm. J. Royal Statistic. Soc. 39, 1–22.

[ref29] Di Martino A. O’connor D. Chen B. Alaerts K. Anderson J. S. Assaf M. . (2017). Enhancing studies of the connectome in autism using the autism brain imaging data exchange II. Scientific Data 4, 1–15. doi: 10.1038/sdata.2017.10 PMC5349246 28291247

[ref30] Dong X. Peng J. Yang Z. Wu X. (2019). Multimodality white matter tract segmentation using CNN.

[ref31] Dosovitskiy A. Beyer L. Kolesnikov A. Weissenborn D. Zhai X. Unterthiner T. . (2020). An image is worth 16x16 words: Transformers for image recognition at scale. arXiv

[ref32] Duclap D. Lebois A. Schmitt B. Riff O. Guevara P. Marrakchi-Kacem L. . (2012). Connectomist-2.0: a novel diffusion analysis toolbox for BrainVISA. In Proceedings of the 29th ESMRMB meeting

[ref33] Dumais F. Legarreta J. H. Lemaire C. Poulin P. Rheault F. Petit L. . (2023). FIESTA: autoencoders for accurate fiber segmentation in tractography. NeuroImage 279:120288. doi: 10.1016/j.neuroimage.2023.120288, PMID: 37495198

[ref34] El Kouby V. Cointepas Y. Poupon C. Riviere D. Golestani N. Poline J. B. . (2005). MR diffusion-based inference of a fiber bundle model from a population of subjects. Med. Image Comput. Comput. Assist. Interv. 8, 196–204. doi: 10.1007/11566465_25, PMID: 16685846

[ref35] Essayed W. I. Zhang F. Unadkat P. Cosgrove G. R. Golby A. J. O'Donnell L. J. (2017). White matter tractography for neurosurgical planning: a topography-based review of the current state of the art. NeuroImage 15, 659–672. doi: 10.1016/j.nicl.2017.06.011, PMID: 28664037 PMC5480983

[ref36] Fischer B. Modersitzki J. (2003). FLIRT: A flexible image registration toolbox. International workshop on biomedical image registration,

[ref37] Frey B. J. Dueck D. (2007). Clustering by passing messages between data points. Science 315, 972–976. doi: 10.1126/science.1136800, PMID: 17218491

[ref38] Froeling M. Tax C. M. Vos S. B. Luijten P. R. Leemans A. (2017). “MASSIVE” brain dataset: multiple acquisitions for standardization of structural imaging validation and evaluation. Magn. Reson. Med. 77, 1797–1809. doi: 10.1002/mrm.26259, PMID: 27173617

[ref39] Fu Y. Lei Y. Wang T. Curran W. J. Liu T. Yang X. (2020). Deep learning in medical image registration: a review. Phys. Med. Biol. 65:20TR01. doi: 10.1088/1361-6560/ab843e, PMID: 32217829 PMC7759388

[ref40] Garyfallidis E. Brett M. Correia M. M. Williams G. B. Nimmo-Smith I. (2012). QuickBundles, a method for Tractography simplification. Front. Neurosci. 6:175. doi: 10.3389/fnins.2012.00175, PMID: 23248578 PMC3518823

[ref41] Garyfallidis E. Côté M.-A. Rheault F. Sidhu J. Hau J. Petit L. . (2018). Recognition of white matter bundles using local and global streamline-based registration and clustering. NeuroImage 170, 283–295. doi: 10.1016/j.neuroimage.2017.07.015, PMID: 28712994

[ref42] Ghazi N. Aarabi M. H. Soltanian-Zadeh H. (2023). Deep learning methods for identification of white matter Fiber tracts: review of state-of-the-art and future prospective. Neuroinformatics 21, 517–548. doi: 10.1007/s12021-023-09636-4 37328715

[ref43] Glasser M. F. Smith S. M. Marcus D. S. Andersson J. L. Auerbach E. J. Behrens T. E. . (2016). The human connectome project's neuroimaging approach. Nat. Neurosci. 19, 1175–1187. doi: 10.1038/nn.4361, PMID: 27571196 PMC6172654

[ref44] Guevara M. Guevara P. Roman C. Mangin J. F. (2020). Superficial white matter: a review on the dMRI analysis methods and applications. NeuroImage 212:116673. doi: 10.1016/j.neuroimage.2020.116673, PMID: 32114152

[ref45] Guevara M. Roman C. Houenou J. Duclap D. Poupon C. Mangin J. F. . (2017). Reproducibility of superficial white matter tracts using diffusion-weighted imaging tractography. NeuroImage 147, 703–725. doi: 10.1016/j.neuroimage.2016.11.066, PMID: 28034765

[ref46] Gupta V. Thomopoulos S. I. Corbin C. K. Rashid F. Thompson P. M. (2018). Fibernet 2.0: an automatic neural network based tool for clustering white matter fibers in the brain. 2018 IEEE 15th International Symposium on Biomedical Imaging (ISBI 2018)

[ref47] Gupta V. Thomopoulos S. I. Rashid F. M. Thompson P. M. (2017). FiberNET: an ensemble deep learning framework for clustering white matter fibers. Medical image computing and computer assisted intervention− MICCAI 2017: 20th International Conference, Quebec City, QC, Canada, September 11–13, 2017, Proceedings, Part I 20

[ref48] Hatamizadeh A. Nath V. Tang Y. Yang D. Roth H. R. Xu D. (2021). Swin unetr: Swin transformers for semantic segmentation of brain tumors in MRI images (pp. 272–284.) Cham: Springer International Publishing.

[ref49] Heker M. Amer R. Alexandroni G. Greenspan H. (2016). Automated supervised segmentation of anatomical fiber tracts using an AdaBoost framework. 2016 IEEE International Conference on the Science of Electrical Engineering (ICSEE)

[ref50] Hofman A. Brusselle G. G. Murad S. D. van Duijn C. M. Franco O. H. Goedegebure A. . (2015). The Rotterdam study: 2016 objectives and design update. Eur. J. Epidemiol. 30, 661–708. doi: 10.1007/s10654-015-0082-x, PMID: 26386597 PMC4579264

[ref51] Huang Z. Wang X. Wei Y. Huang L. Shi H. Liu W. . (2023). CCNet: Criss-cross attention for semantic segmentation. IEEE Trans. Pattern Anal. Mach. Intell. 45, 6896–6908. doi: 10.1109/TPAMI.2020.3007032, PMID: 32750802

[ref52] Huang H. Zhang J. Wakana S. Zhang W. Ren T. Richards L. J. . (2006). White and gray matter development in human fetal, newborn and pediatric brains. NeuroImage 33, 27–38. doi: 10.1016/j.neuroimage.2006.06.009, PMID: 16905335

[ref53] Ille S. Ohlerth A.-K. Colle D. Colle H. Dragoy O. Goodden J. . (2021). Augmented reality for the virtual dissection of white matter pathways. Acta Neurochir. 163, 895–903. doi: 10.1007/s00701-020-04545-w, PMID: 33026532 PMC7966623

[ref54] Isensee F. Jaeger P. F. Kohl S. A. A. Petersen J. Maier-Hein K. H. (2021). nnU-net: a self-configuring method for deep learning-based biomedical image segmentation. Nat. Methods 18, 203–211. doi: 10.1038/s41592-020-01008-z, PMID: 33288961

[ref55] Jeurissen B. Descoteaux M. Mori S. Leemans A. (2019). Diffusion MRI fiber tractography of the brain. NMR Biomed. 32:e3785. doi: 10.1002/nbm.3785 28945294

[ref56] Jin Y. Cetingül H. E. (2015). Tractography-embedded white matter stream clustering. 2015 IEEE 12th International Symposium on Biomedical Imaging (ISBI)

[ref57] Jin Y. Shi Y. Zhan L. De Zubicaray G. I. McMahon K. L. Martin N. G. . (2013). Labeling white matter tracts in HARDI by fusing multiple tract atlases with applications to genetics. 2013 IEEE 10th International Symposium on Biomedical Imaging 10.1109/ISBI.2013.6556524 PMC4236723 25419442

[ref58] Jordan K. M. Lauricella M. Licata A. E. Sacco S. Asteggiano C. Wang C. . (2021). Cortically constrained shape recognition: automated white matter tract segmentation validated in the pediatric brain. J. Neuroimaging 31, 758–772. doi: 10.1111/jon.12854, PMID: 33878229 PMC12057640

[ref59] Kamali T. Stashuk D. (2016). Automated segmentation of white matter fiber bundles using diffusion tensor imaging data and a new density based clustering algorithm. Artif. Intell. Med. 73, 14–22. doi: 10.1016/j.artmed.2016.09.003, PMID: 27926378

[ref60] Kamnitsas K. Ledig C. Newcombe V. F. Simpson J. P. Kane A. D. Menon D. K. . (2017). Efficient multi-scale 3D CNN with fully connected CRF for accurate brain lesion segmentation. Med. Image Anal. 36, 61–78. doi: 10.1016/j.media.2016.10.004, PMID: 27865153

[ref61] Kumar K. Desrosiers C. (2016). A sparse coding approach for the efficient representation and segmentation of white matter fibers. 2016 IEEE 13th International Symposium on Biomedical Imaging (ISBI)

[ref62] Labra N. Guevara P. Duclap D. Houenou J. Poupon C. Mangin J.-F. . (2017). Fast automatic segmentation of white matter streamlines based on a multi-subject bundle atlas. Neuroinformatics 15, 71–86. doi: 10.1007/s12021-016-9316-7, PMID: 27722821

[ref63] Lacante M. Van Esbroeck R. De Vos A. (2008). Met een dynamische keuzebegeleiding naar een effectieve keuzebekwaamheid: Eindrapport OBPWO projecten 04.01 & 02.02 en Ministerieel Initiatief. Brussel/Leuven: Vrije Universiteit Brussel/Katholieke Universiteit Leuven.

[ref64] Lazar M. Alexander A. Thottakara P. Badie B. Field A. (2006). White matter reorganization after surgical resection of brain tumors and vascular malformations. Am. J. Neuroradiol. 27, 1258–1271. PMID: 16775277 PMC8133916

[ref65] Le Bihan D. Johansen-Berg H. (2012). Diffusion MRI at 25: exploring brain tissue structure and function. NeuroImage 61, 324–341. doi: 10.1016/j.neuroimage.2011.11.006, PMID: 22120012 PMC3683822

[ref66] Li S. Chen Z. Guo W. Zeng Q. Feng Y. (2021) Two parallel stages deep learning network for anterior visual pathway segmentation. Computational Diffusion MRI, International MICCAI Workshop: Lima, Peru

[ref67] Li B. De Groot M. Steketee R. M. Meijboom R. Smits M. Vernooij M. W. . (2020). Neuro4Neuro: a neural network approach for neural tract segmentation using large-scale population-based diffusion imaging. NeuroImage 218:116993. doi: 10.1016/j.neuroimage.2020.116993, PMID: 32492510

[ref68] Liu F. Feng J. Chen G. Wu Y. Hong Y. Yap P. T. . (2019). DeepBundle: Fiber bundle Parcellation with graph convolution neural networks. Graph. Learn. Med. Imaging 11849, 88–95. doi: 10.1007/978-3-030-35817-4_11, PMID: 34485996 PMC8411944

[ref69] Liu W. Lu Q. Zhuo Z. Li Y. Duan Y. Yu P. . (2022). Volumetric segmentation of white matter tracts with label embedding. NeuroImage 250:118934. doi: 10.1016/j.neuroimage.2022.118934, PMID: 35091078

[ref70] Liu W. Zhuo Z. Liu Y. Ye C. (2023). One-shot segmentation of novel white matter tracts via extensive data augmentation and adaptive knowledge transfer. Med. Image Anal. 90:102968. doi: 10.1016/j.media.2023.102968, PMID: 37729793

[ref71] Logiraj K. Sotheeswaran S. Jeyasuthan M. Ratnarajah N. (2021a). Clustering of major white matter bundles using tract-specific geometric curve features. 2021 10th International Conference on Information and Automation for Sustainability (ICIAfS)

[ref72] Logiraj K. Thanikasalam K. Sotheeswaran S. Ratnarajah N. (2021b). TractNet: a deep learning approach on 3D curves for segmenting white matter fibre bundles. 2021 21st International Conference on Advances in ICT for Emerging Regions (ICter)

[ref73] Lu Q. Li Y. Ye C. (2020). White matter tract segmentation with self-supervised learning. Medical image computing and computer assisted intervention–MICCAI 2020: 23rd International Conference, Lima, Peru, October 4–8, 2020, Proceedings, Part VII 23

[ref74] Lu Q. Li Y. Ye C. (2021). Volumetric white matter tract segmentation with nested self-supervised learning using sequential pretext tasks. Med. Image Anal. 72:102094. doi: 10.1016/j.media.2021.102094, PMID: 34004493

[ref75] Lu Q. Liu W. Zhuo Z. Li Y. Duan Y. Yu P. . (2022). A transfer learning approach to few-shot segmentation of novel white matter tracts. Med. Image Anal. 79:102454. doi: 10.1016/j.media.2022.102454, PMID: 35468555

[ref76] Lucena O. Borges P. Cardoso J. Ashkan K. Sparks R. Ourselin S. (2022). Informative and reliable tract segmentation for preoperative planning. Front. Radiol. 2:866974. doi: 10.3389/fradi.2022.866974, PMID: 37492653 PMC10365092

[ref77] Makropoulos A. Robinson E. C. Schuh A. Wright R. Fitzgibbon S. Bozek J. . (2018). The developing human connectome project: a minimal processing pipeline for neonatal cortical surface reconstruction. NeuroImage 173, 88–112. doi: 10.1016/j.neuroimage.2018.01.054, PMID: 29409960 PMC6783314

[ref78] Mancini M. Vos S. B. Vakharia V. N. O'Keeffe A. G. Trimmel K. Barkhof F. . (2019). Automated fiber tract reconstruction for surgery planning: extensive validation in language-related white matter tracts. Neuroimage Clin. 23:101883. doi: 10.1016/j.nicl.2019.101883, PMID: 31163386 PMC6545442

[ref79] Marek K. Jennings D. Lasch S. Siderowf A. Tanner C. Simuni T. . (2011). The Parkinson progression marker initiative (PPMI). Prog. Neurobiol. 95, 629–635. doi: 10.1016/j.pneurobio.2011.09.005, PMID: 21930184 PMC9014725

[ref80] Mazoyer B. Mellet E. Perchey G. Zago L. Crivello F. Jobard G. . (2016). BIL&GIN: a neuroimaging, cognitive, behavioral, and genetic database for the study of human brain lateralization. NeuroImage 124, 1225–1231. doi: 10.1016/j.neuroimage.2015.02.071 25840118

[ref81] Milletari F. Navab N. Ahmadi S.-A. (2016). V-net: fully convolutional neural networks for volumetric medical image segmentation. 2016 fourth international conference on 3D vision (3DV),

[ref82] Mori S. Crain B. J. Chacko V. P. Van Zijl P. C. (1999). Three-dimensional tracking of axonal projections in the brain by magnetic resonance imaging. Ann. Neurol. 45, 265–269. doi: 10.1002/1531-8249(199902)45:2<265::AID-ANA21>3.0.CO;2-3, PMID: 9989633

[ref83] Mori S. van Zijl P. (2007). Human white matter atlas. Am. J. Psychiatry 164:1005. doi: 10.1176/ajp.2007.164.7.1005 17606649

[ref84] Moulavi D. Jaskowiak P. A. Campello R. J. Zimek A. Sander J. (2014). Density-based clustering validation. Proceedings of the 2014 SIAM international conference on data mining

[ref85] Nelkenbaum I. Tsarfaty G. Kiryati N. Konen E. Mayer A. (2020). Automatic segmentation of white matter tracts using multiple brain MRI sequences. 2020 IEEE 17th International Symposium on Biomedical Imaging (ISBI)

[ref86] Ngattai Lam P. D. Belhomme G. Ferrall J. Patterson B. Styner M. Prieto J. C. (2018). TRAFIC: Fiber tract classification using deep learning. Proc. SPIE Int. Soc. Opt. Eng. 10574:1057412. doi: 10.1117/12.2293931, PMID: 29780197 PMC5956534

[ref87] O’Donnell L. J. Suter Y. Rigolo L. Kahali P. Zhang F. Norton I. . (2017). Automated white matter fiber tract identification in patients with brain tumors. NeuroImage 13, 138–153. doi: 10.1016/j.nicl.2016.11.023, PMID: 27981029 PMC5144756

[ref88] Ocegueda O. Rivera M. (2013). Multi-tensor Field spectral segmentation for white matter fiber bundle classification. 2013 IEEE 10th International Symposium on Biomedical Imaging

[ref89] O'Donnell L. J. Westin C.-F. (2007). Automatic tractography segmentation using a high-dimensional white matter atlas. IEEE Trans. Med. Imaging 26, 1562–1575. doi: 10.1109/TMI.2007.906785, PMID: 18041271

[ref90] Oishi K. Faria A. Jiang H. Li X. Akhter K. Zhang J. . (2009). Atlas-based whole brain white matter analysis using large deformation diffeomorphic metric mapping: application to normal elderly and Alzheimer's disease participants. NeuroImage 46, 486–499. doi: 10.1016/j.neuroimage.2009.01.002, PMID: 19385016 PMC2885858

[ref91] Oishi K. Mori S. Donohue P. K. Ernst T. Anderson L. Buchthal S. . (2011). Multi-contrast human neonatal brain atlas: application to normal neonate development analysis. NeuroImage 56, 8–20. doi: 10.1016/j.neuroimage.2011.01.051, PMID: 21276861 PMC3066278

[ref92] Oliveira F. P. Tavares J. M. R. (2014). Medical image registration: a review. Comput. Methods Biomech. Biomed. Engin. 17, 73–93. doi: 10.1080/10255842.2012.670855 22435355

[ref93] Peretzke R. Maier-Hein K. H. Bohn J. Kirchhoff Y. Roy S. Oberli-Palma S. . (2023). atTRACTive: semi-automatic white matter tract segmentation using active learning. International Conference on Medical Image Computing and Computer-Assisted Intervention

[ref94] Poldrack R. A. Congdon E. Triplett W. Gorgolewski K. Karlsgodt K. Mumford J. . (2016). A phenome-wide examination of neural and cognitive function. Scientific Data 3, 1–12. doi: 10.1038/sdata.2016.110 PMC5139672 27922632

[ref95] Pomiecko K. Sestili C. Fissell K. Pathak S. Okonkwo D. Schneider W. (2019). 3D convolutional neural network segmentation of white matter tract masks from MR diffusion anisotropy maps. 2019 IEEE 16th International Symposium on Biomedical Imaging (ISBI 2019)

[ref96] Poulin P. Jorgens D. Jodoin P. M. Descoteaux M. (2019). Tractography and machine learning: current state and open challenges. Magn. Reson. Imaging 64, 37–48. doi: 10.1016/j.mri.2019.04.013, PMID: 31078615

[ref97] Poulin P. Theaud G. Rheault F. St-Onge E. Bore A. Renauld E. . (2022). TractoInferno-A large-scale, open-source, multi-site database for machine learning dMRI tractography. Scientific Data 9:725. doi: 10.1038/s41597-022-01833-1, PMID: 36433966 PMC9700736

[ref98] Poupon C. Rieul B. Kezele I. Perrin M. Poupon F. Mangin J. F. (2008). New diffusion phantoms dedicated to the study and validation of high-angular-resolution diffusion imaging (HARDI) models. Magnet. Reson. Med 60, 1276–1283. doi: 10.1002/mrm.21789, PMID: 19030160

[ref99] Pujol S. Wells W. Pierpaoli C. Brun C. Gee J. Cheng G. . (2015). The DTI challenge: toward standardized evaluation of diffusion tensor imaging Tractography for neurosurgery. J. Neuroimaging 25, 875–882. doi: 10.1111/jon.12283, PMID: 26259925 PMC4641305

[ref100] Qi C. R. Su H. Mo K. Guibas L. J. (2017). Pointnet: deep learning on point sets for 3d classification and segmentation. Proceedings of the IEEE conference on computer vision and pattern recognition

[ref101] Radwan A. M. Sunaert S. Schilling K. Descoteaux M. Landman B. A. Vandenbulcke M. . (2022). An atlas of white matter anatomy, its variability, and reproducibility based on constrained spherical deconvolution of diffusion MRI. NeuroImage 254:119029. doi: 10.1016/j.neuroimage.2022.119029, PMID: 35231632 PMC10265547

[ref102] Ratnarajah N. Qiu A. (2014). Multi-label segmentation of white matter structures: application to neonatal brains. NeuroImage 102, 913–922. doi: 10.1016/j.neuroimage.2014.08.001, PMID: 25111473

[ref103] Reynolds D. A. Quatieri T. F. Dunn R. B. (2000). Speaker verification using adapted Gaussian mixture models. Digit. Signal Process. 10, 19–41. doi: 10.1006/dspr.1999.0361

[ref104] Rheault F. De Benedictis A. Daducci A. Maffei C. Tax C. M. Romascano D. . (2020). Tractostorm: the what, why, and how of tractography dissection reproducibility. Hum. Brain Mapp. 41, 1859–1874. doi: 10.1002/hbm.24917, PMID: 31925871 PMC7267902

[ref105] Rheault F. Schilling K. G. Obaid S. Begnoche J. P. Cutting L. E. Descoteaux M. . (2022a). The influence of regions of interest on tractography virtual dissection protocols: general principles to learn and to follow. Brain Struct. Funct. 227, 2191–2207. doi: 10.1007/s00429-022-02518-6 35672532 PMC9884471

[ref106] Rheault F. Schilling K. G. Valcourt-Caron A. Théberge A. Poirier C. Grenier G. . (2022b). Tractostorm 2: optimizing tractography dissection reproducibility with segmentation protocol dissemination. Hum. Brain Mapp. 43, 2134–2147. doi: 10.1002/hbm.25777, PMID: 35141980 PMC8996349

[ref107] Roman C. Guevara M. Valenzuela R. Figueroa M. Houenou J. Duclap D. . (2017). Clustering of whole-brain white matter short association bundles using HARDI data. Front. Neuroinform. 11:73. doi: 10.3389/fninf.2017.00073, PMID: 29311886 PMC5744462

[ref108] Ronneberger O. Fischer P. Brox T. (2015). U-net: convolutional networks for biomedical image segmentation. Medical image computing and computer-assisted intervention–MICCAI 2015. 18th International Conference, Munich, Germany, October 5–9, 2015, Proceedings, Part III 18

[ref109] Sadeghi N. Prastawa M. Fletcher P. T. Wolff J. Gilmore J. H. Gerig G. (2013). Regional characterization of longitudinal DT-MRI to study white matter maturation of the early developing brain. NeuroImage 68, 236–247. doi: 10.1016/j.neuroimage.2012.11.040, PMID: 23235270 PMC3693970

[ref110] Schilling K. G. Rheault F. Petit L. Hansen C. B. Nath V. Yeh F.-C. . (2021). Tractography dissection variability: what happens when 42 groups dissect 14 white matter bundles on the same dataset? NeuroImage 243:118502. doi: 10.1016/j.neuroimage.2021.118502, PMID: 34433094 PMC8855321

[ref111] Schmitt B. Lebois A. Duclap D. Guevara P. Poupon F. Rivière D. . (2012). CONNECT/ARCHI: an open database to infer atlases of the human brain connectivity. ESMRMB 272:2012.

[ref112] Sharmin N. Olivetti E. Avesani P. (2018). White matter tract segmentation as multiple linear assignment problems. Front. Neurosci. 11:754. doi: 10.3389/fnins.2017.00754, PMID: 29467600 PMC5808221

[ref113] Shi J. Malik J. (2000). Normalized cuts and image segmentation. IEEE Trans. Pattern Anal. Mach. Intell. 22, 888–905. doi: 10.1109/34.868688

[ref114] Siless V. Chang K. Fischl B. Yendiki A. (2018). AnatomiCuts: hierarchical clustering of tractography streamlines based on anatomical similarity. NeuroImage 166, 32–45. doi: 10.1016/j.neuroimage.2017.10.058, PMID: 29100937 PMC6152885

[ref115] Soh S.-E. Tint M. T. Gluckman P. D. Godfrey K. M. Rifkin-Graboi A. Chan Y. H. . (2014). Cohort profile: growing up in Singapore towards healthy outcomes (GUSTO) birth cohort study. Int. J. Epidemiol. 43, 1401–1409. doi: 10.1093/ije/dyt125, PMID: 23912809

[ref116] Steketee R. M. Bron E. E. Meijboom R. Houston G. C. Klein S. Mutsaerts H. J. . (2016). Early-stage differentiation between presenile Alzheimer’s disease and frontotemporal dementia using arterial spin labeling MRI. Eur. Radiol. 26, 244–253. doi: 10.1007/s00330-015-3789-x, PMID: 26024845 PMC4666273

[ref117] Teeuw J. Caan M. W. Olabarriaga S. D. (2015). Robust automated white matter pathway reconstruction for large studies. Medical image computing and computer-assisted intervention--MICCAI 2015, 18th International Conference, Munich, Germany, October 5–9, 2015, Proceedings, Part I 18

[ref118] Toga A. W. Mazziotta J. C. (2002). Brain mapping: the methods, vol. 1 Academic Press.

[ref119] Tunc B. Parker W. A. Ingalhalikar M. Verma R. (2014). Automated tract extraction via atlas based adaptive clustering. NeuroImage 102, 596–607. doi: 10.1016/j.neuroimage.2014.08.021, PMID: 25134977 PMC4252913

[ref120] Ugurlu D. Firat Z. Ture U. Unal G. (2019). Supervised classification of white matter fibers based on neighborhood fiber orientation distributions using an ensemble of neural networks. Comput. Diffusion MRI 2018:22. doi: 10.1007/978-3-030-05831-9_12

[ref121] Van Essen D. C. Smith S. M. Barch D. M. Behrens T. E. Yacoub E. Ugurbil K. . (2013). The WU-Minn human connectome project: an overview. NeuroImage 80, 62–79. doi: 10.1016/j.neuroimage.2013.05.041 23684880 PMC3724347

[ref122] Van Essen D. C. Ugurbil K. Auerbach E. Barch D. Behrens T. E. Bucholz R. . (2012). The human connectome project: a data acquisition perspective. NeuroImage 62, 2222–2231. doi: 10.1016/j.neuroimage.2012.02.018, PMID: 22366334 PMC3606888

[ref123] Vanderweyen D. C. Theaud G. Sidhu J. Rheault F. Sarubbo S. Descoteaux M. . (2020). The role of diffusion tractography in refining glial tumor resection. Brain Struct. Funct. 225, 1413–1436. doi: 10.1007/s00429-020-02056-z, PMID: 32180019

[ref124] Vaswani A. Shazeer N. Parmar N. Uszkoreit J. Jones L. Gomez A. N. . (2017). Attention is all you need. Adv. Neural Inf. Proces. Syst. 30

[ref125] Vázquez A. López-López N. Labra N. Figueroa M. Poupon C. Mangin J.-F. . (2019). Parallel optimization of fiber bundle segmentation for massive tractography datasets. 2019 IEEE 16th International Symposium on Biomedical Imaging (ISBI 2019)

[ref126] Vázquez A. López-López N. Sánchez A. Houenou J. Poupon C. Mangin J.-F. . (2020). FFClust: fast fiber clustering for large tractography datasets for a detailed study of brain connectivity. NeuroImage 220:117070. doi: 10.1016/j.neuroimage.2020.117070, PMID: 32599269

[ref127] Viola P. Jones M. (2001). Rapid object detection using a boosted cascade of simple features. In Proceedings of the 2001 IEEE computer society conference on computer vision and pattern recognition. CVPR 2001

[ref128] Volkow N. D. Koob G. F. Croyle R. T. Bianchi D. W. Gordon J. A. Koroshetz W. J. . (2018). The conception of the ABCD study: from substance use to a broad NIH collaboration. Dev. Cogn. Neurosci. 32, 4–7. doi: 10.1016/j.dcn.2017.10.002, PMID: 29051027 PMC5893417

[ref129] Vu T. H. Monga V. (2017). Fast low-rank shared dictionary learning for image classification. IEEE Trans. Image Process. 26, 5160–5175. doi: 10.1109/TIP.2017.2729885, PMID: 28742035

[ref130] Wakana S. Caprihan A. Panzenboeck M. M. Fallon J. H. Perry M. Gollub R. L. . (2007). Reproducibility of quantitative tractography methods applied to cerebral white matter. NeuroImage 36, 630–644. doi: 10.1016/j.neuroimage.2007.02.049, PMID: 17481925 PMC2350213

[ref131] Wang Z. Lv Y. He M. Ge E. Qiang N. Ge B. (2022). Accurate corresponding Fiber tract segmentation via FiberGeoMap learner. International Conference on Medical Image Computing and Computer-Assisted Intervention

[ref132] Warrington S. Bryant K. L. Khrapitchev A. A. Sallet J. Charquero-Ballester M. Douaud G. . (2020). XTRACT-standardised protocols for automated tractography in the human and macaque brain. NeuroImage 217:116923. doi: 10.1016/j.neuroimage.2020.116923, PMID: 32407993 PMC7260058

[ref133] Wassermann D. Makris N. Rathi Y. Shenton M. Kikinis R. Kubicki M. . (2013). On describing human white matter anatomy: the white matter query language. Med. Image Comput. Comput. Assist. Interv. 16, 647–654. doi: 10.1007/978-3-642-40811-3_81, PMID: 24505722 PMC4029160

[ref134] Wassermann D. Makris N. Rathi Y. Shenton M. Kikinis R. Kubicki M. . (2016). The white matter query language: a novel approach for describing human white matter anatomy. Brain Struct. Funct. 221, 4705–4721. doi: 10.1007/s00429-015-1179-4 26754839 PMC4940319

[ref135] Wasserthal J. Neher P. F. Hirjak D. Maier-Hein K. H. (2019). Combined tract segmentation and orientation mapping for bundle-specific tractography. Med. Image Anal. 58:101559. doi: 10.1016/j.media.2019.101559, PMID: 31542711

[ref136] Wasserthal J. Neher P. Maier-Hein K. H. (2018). TractSeg - fast and accurate white matter tract segmentation. NeuroImage 183, 239–253. doi: 10.1016/j.neuroimage.2018.07.070, PMID: 30086412

[ref137] Wu Y. Hong Y. Ahmad S. Lin W. Shen D. Yap P. T. . (2020). Tract dictionary learning for fast and robust recognition of Fiber bundles. Med. Image Comput. Comput. Assist. Interv. 12267, 251–259. doi: 10.1007/978-3-030-59728-3_25, PMID: 34195699 PMC8238464

[ref138] Xiao H. Li L. Liu Q. Zhu X. Zhang Q. (2023). Transformers in medical image segmentation: a review. Biomed. Signal Process. Control 84:104791. doi: 10.1016/j.bspc.2023.104791

[ref139] Xu C. Sun G. Liang R. Xu X. (2021). Vector field streamline clustering framework for brain fiber tract segmentation. IEEE Transac. Cognit. Develop. Syst. 14, 1066–1081. doi: 10.1109/TCDS.2021.3094555

[ref140] Xu H. Xue T. Liu D. Zhang F. Westin C.-F. Kikinis R. . (2023). A registration-and uncertainty-based framework for white matter tract segmentation with only one annotated subject. 2023 IEEE 20th International Symposium on Biomedical Imaging (ISBI)

[ref141] Xue T. Zhang F. Zhang C. Chen Y. Song Y. Golby A. J. . (2023). Superficial white matter analysis: an efficient point-cloud-based deep learning framework with supervised contrastive learning for consistent tractography parcellation across populations and dMRI acquisitions. Med. Image Anal. 85:102759. doi: 10.1016/j.media.2023.102759, PMID: 36706638 PMC9975054

[ref142] Yamada K. Sakai K. Akazawa K. Yuen S. Nishimura T. (2009). MR tractography: a review of its clinical applications. Magn. Reson. Med. Sci. 8, 165–174. doi: 10.2463/mrms.8.165, PMID: 20035125

[ref143] Yang Z. Li X. Zhou J. Wu X. Ding Z. (2020). Functional clustering of whole brain white matter fibers. J. Neurosci. Methods 335:108626. doi: 10.1016/j.jneumeth.2020.108626, PMID: 32032716 PMC7093303

[ref144] Yeatman J. D. Dougherty R. F. Myall N. J. Wandell B. A. Feldman H. M. (2012). Tract profiles of white matter properties: automating fiber-tract quantification. PLoS One 7:e49790. doi: 10.1371/journal.pone.0049790, PMID: 23166771 PMC3498174

[ref145] Yendiki A. Panneck P. Srinivasan P. Stevens A. Zollei L. Augustinack J. . (2011). Automated probabilistic reconstruction of white-matter pathways in health and disease using an atlas of the underlying anatomy. Front. Neuroinform. 5:23. doi: 10.3389/fninf.2011.00023, PMID: 22016733 PMC3193073

[ref146] Yin H. Xu P. Cui H. Chen G. Ma J. (2022). DC 2 U-net: tract segmentation in brain white matter using dense Criss-cross U-net. International Workshop on Computational Diffusion MRI

[ref147] Yoo S. W. Guevara P. Jeong Y. Yoo K. Shin J. S. Mangin J.-F. . (2015). An example-based multi-atlas approach to automatic labeling of white matter tracts. PLoS One 10:e0133337. doi: 10.1371/journal.pone.0133337, PMID: 26225419 PMC4520495

[ref148] Yun S. Han D. Oh S. J. Chun S. Choe J. Yoo Y. (2019). Cutmix: regularization strategy to train strong classifiers with localizable features. Proceedings of the IEEE/CVF international conference on computer vision

[ref149] Yushkevich P. A. Zhang H. Simon T. J. Gee J. C. (2008). Structure-specific statistical mapping of white matter tracts. NeuroImage 41, 448–461. doi: 10.1016/j.neuroimage.2008.01.013 18407524 PMC2519052

[ref150] Zhang H. Cisse M. Dauphin Y. N. Lopez-Paz D. (2017). mixup: Beyond empirical risk minimization. arXiv preprint arXiv:1710.09412

[ref151] Zhang F. Daducci A. He Y. Schiavi S. Seguin C. Smith R. E. . (2022). Quantitative mapping of the brain’s structural connectivity using diffusion MRI tractography: a review. NeuroImage 249:118870. doi: 10.1016/j.neuroimage.2021.118870, PMID: 34979249 PMC9257891

[ref152] Zhang F. Hoffmann N. Karayumak S. C. Rathi Y. Golby A. J. O’Donnell L. J. (2019). Deep white matter analysis: fast, consistent tractography segmentation across populations and dMRI acquisitions. Int. Conference Med. Image Comput. Comput. Assist. Intervent. doi: 10.1007/978-3-030-32248-9_67 PMC7301958 32558816

[ref153] Zhang F. Karayumak S. C. Hoffmann N. Rathi Y. Golby A. J. O’Donnell L. J. (2020). Deep white matter analysis (DeepWMA): fast and consistent tractography segmentation. Med. Image Anal. 65:101761. doi: 10.1016/j.media.2020.101761, PMID: 32622304 PMC7483951

[ref154] Zhang F. Wells W. M. O’Donnell L. J. (2021). Deep diffusion MRI registration (DDMReg): a deep learning method for diffusion MRI registration. IEEE Trans. Med. Imaging 41, 1454–1467. doi: 10.1109/TMI.2021.3139507 PMC9273049 34968177

[ref155] Zhang F. Wu Y. Norton I. Rigolo L. Rathi Y. Makris N. . (2018). An anatomically curated fiber clustering white matter atlas for consistent white matter tract parcellation across the lifespan. NeuroImage 179, 429–447. doi: 10.1016/j.neuroimage.2018.06.027, PMID: 29920375 PMC6080311

[ref156] Zhang Y. Zhang J. Oishi K. Faria A. V. Jiang H. Li X. . (2010). Atlas-guided tract reconstruction for automated and comprehensive examination of the white matter anatomy. NeuroImage 52, 1289–1301. doi: 10.1016/j.neuroimage.2010.05.049, PMID: 20570617 PMC2910162

[ref157] Zhao Y. Su J. Yang Z. Ding Z. (2022). A Riemannian framework for functional clustering of whole brain white matter fibers. 2022 IEEE 19th International Symposium on Biomedical Imaging (ISBI)

